# Isolation, Characterization and Genomic Analysis of a Novel Bacteriophage VB_EcoS-Golestan Infecting Multidrug-Resistant *Escherichia coli* Isolated from Urinary Tract Infection

**DOI:** 10.1038/s41598-020-63048-x

**Published:** 2020-05-06

**Authors:** Mahsa Yazdi, Majid Bouzari, Ezzat Allah Ghaemi, Khashayar Shahin

**Affiliations:** 1 Department of Biology, Faculty of Sciences, University of Isfahan, 81746-73441 Isfahan, Iran; 2 Laboratory Sciences Research Center, Golestan University of Medical Sciences, 4934174515 Gorgan, Iran

**Keywords:** Bacteriophages, Bacteriophages, Urogenital diseases, Urogenital diseases

## Abstract

*Escherichia coli* (*E. coli*) is one of the most common uropathogenic bacteria. The emergence of multi-drug resistance among these bacteria resulted in a worldwide public health problem which requires alternative treatment approaches such as phage therapy. In this study, phage VB_EcoS-Golestan, a member of *Siphoviridae* family, with high lytic ability against *E. coli* isolates, was isolated from wastewater. Its burst size was large and about 100 plaque-forming units/infected cell, rapid adsorption time, and high resistance to a broad range of pH and temperatures. Bioinformatics analysis of the genomic sequence suggests that VB_EcoS-Golestan is a new phage closely related to *Escherichia* phages in the *Kagunavirus* genus, *Guernseyvirinae* subfamily of *Siphoviridae*. The genome size was 44829 bp bp that encodes 78 putative ORFs, no tRNAs, 7 potential promoter sequences and 13 Rho-factor-independent terminators. No lysogenic mediated genes were detected in VB_EcoS-Golestan genome. Overall VB_EcoS-Golestan might be used as a potential treatment approach for controlling *E. coli* mediated urinary tract infection, however, further studies are essential to ensure its safety.

## Introduction

*E. coli* is one of the most common pathogens causing urinary tract infection.Uropathogenic strains of *Escherichia coli* (UPECs) account for 70–95% of community-acquired UTIs and over 50% of nosocomial UTIs^1–4^. UPECs expresses several virulence factors to break the inertia of the mucosal barrier. An important virulence factor of these bacteria is their ability to adhere to the host epithelial cells, which is a precondition for the creation of infectious diseases, mostly through the expression of fimbriae^5,6^. Emergence of antibiotic resistance among UPEC strains can occur through several mechanisms, and has been reported frequently over the last decade. This made them a worldwide concern as it significantly reduced the efficiency of the first-line treatment agents (like ampicillin, nitrofurantoin, sulfamethoxazole/trimethoprim (SXT) and fluoroquinolones) against this pathogen^7^.

Emergences of such multi-drug resistant (MDR) strains limits the treatment options^3,8–11^. Therefore, finding new effective strategies for treatment of MDR UPECs is a must^7^. Nowadays, phage therapy, once more, has become a hopeful biological method to manage antibiotic-resistant infections. This is as a result of the increased knowledge on biology of phage, very high specificity toward their host bacteria, no effect on the normal microflora, and replication and exponential growth in presence of their specific hosts at the infection site^12,13^. Since phage-therapy is still an under-study therapeutic approach, further development of this method requires biological characterization of bacteriophages such as their host specificity, genome diversity, and adaption to their bacterial hosts^14,15^. The current study aimed for comprehensive biological and genomic characterizations of a new lytic bacteriophage against uropathogenic *E. coli*.

## Results and discussion

### Morphology of phage VB_EcoS-Golestan

**Figure 1:**
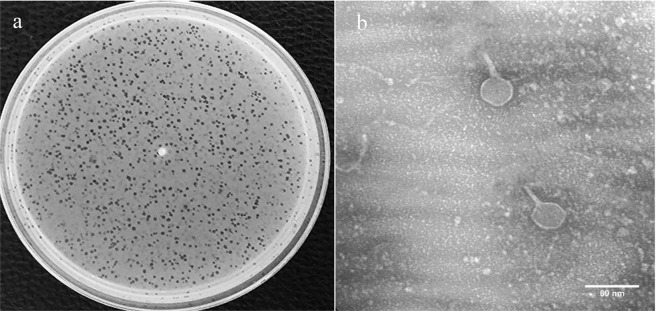
Plaques of phage VB_EcoS-Golestan on the lawn of *E. coli* 333 isolate (**a**). Transmission electron micrograph of the phage (**b**).

Phage VB_EcoS-Golestan produced large, clear and round plaques, with a range of sizes of 1.5–2 mm in diameter on a lawn culture of multidrug resistance UPEC isolate 333 (as a host for VB_EcoS-Golestan) (Fig. 1a). TEM morphology analysis of the phage showed that the phage had a hexagonal capsid with an estimated diameter of 35 ± 0.1 nm, and a noncontractile tail which was 28 ± 0.9 nm long and 7 ± 0.9 nm in diameter (Fig. 1b) (**see** Supplementary Fig. S1**)**. Over 95% of the reported phages belong to *Caudovirales* in which about 62% are phages with non-contractile and lengthy tails belong to *Siphoviridae* family^14,16^. Therefore, based on the morphological features and criterions of the International Committee on Taxonomy of Viruses (ICTV)^17^, VB_EcoS-Golestan was classified within *Caudovirales* order, *Siphoviridae* family.

### Antimicrobial resistance of *E. coli* clinical isolates and host range

Antibiotic resistance pattern of 52 *E. coli* isolates, isolated from infection of urinary tract, is shown in Table 1. Forty one of the isolates (78.8%) were MDR. In agreement with previous studies, this signifies that there is an elevated frequency of antimicrobial resistance among uropathogenic *E. coli*^18,19^. In general, due to the production of β-lactamase enzymes in UPEC isolates, such pathogens have a high resistance to beta-lactam antibiotics which are a common choice for treatment of urinary tract infection^4^ (Supplementary Table S1). In addition, quinolones such as ciprofloxacin, levofloxacin, and ofloxacin are considered as the first-line agents for treating urinary tract infections^20^. The highest resistance among the isolated *E. coli* in this study was observed for ampicillin (92.3%). Moreover, high resistance to ofloxacin and ciprofloxacin (61.5%) was also observed (Supplementary Table S1). All the isolates were sensitive to imipenem, which is one of the choice drugs in MDR UPEC in our region. Recently, resistance to this drug has been reported which limits its use as a drug of choice in the future^21–23^. Observing such a high antibiotic resistance among the isolated UPECs in this study is in agreement with the worldwide emergence of MDR among UPECs. This observation was the driving force of the current research for using bacteriophages as a mean for prevention and control of bacterial infections and further spread of multi-drug resistant strains.

**Table 1:** List of UPEC isolates, their antimicrobial resistance pattern, virulence factors, and sensitivity to phage VB_EcoS-Golestan.

No.	*Escherichia coli* isolates codes	Names and number of antibiotics to which isolates were resistant	Virulence factors	Infection
1	*354 M*	AZT,OFL,AMI,AMC,AMP,CXM,CAZ,FEP,CIPR,CFZ,SAM,CTR,CFM,CTX,GEN(15)	*Fim*	−
2	*5SA*	OFL,AMC,AMP,SXT,CIPR,CFZ,CFM,GEN(8)	*Fim*	−
3	*409 M*	AMP(1)	*Fim,pap,sfa*	−
4	*159 M*	OFL,AMP,CXM,SXT,CIPR,CFZ,CTR,CFM,CTX(9)	*Fim*	−
5	*140 M*	AMP,SXT,SAM(3)	*Fim,pap,sfa*	−
6	*417 M*	AMC,AMP,CXM,SXT,CFZ,CFM,SAM(7)	*Fim,pap,sfa*	−
7	*1–496 M*	AZT,OFL,AMP,CXM,SXT,FEP,CIPR,CFZ,SAM,CTR,CFM,CTX(12)	*Fim,pap,sfa*	+
8	*32 M*	OFL,AMP,SXT,CIPR,SAM,GEN(6)	*Fim,pap,sfa*	+
9	*477B*	AZT,OFL,AMP,CXM,SXT,FEP,CIPR,CFZ,SAM,CTR,CFM,CTX,GEN(13)	*Fim,pap,sfa*	+
10	*1192 M*	AZT,OFL,AMP,SXT,CIPR,CFM(6)	*Fim,pap,sfa*	−
11	*134 M*	AZT,OFL,AMP,CXM,SXT,CAZ,FEP,CIPR,CFZ,SAM,CTR,CFM,CTX,GEN(14)	*Pap,sfa*	−
12	*200 M*	AMP,SXT(2)	*Fim,pap*	−
13	*M-332M*	AZT,OFL,AMP,CXM,SXT,CAZ,FEP,CIPR,CFZ,SAM,CTR,CFM,CTX,GEN(14)	*Fim,pap,sfa*	+
14	*137 M*	AZT,OFL,AMP,CXM,SXT,CAZ,FEP,CIPR,CFZ,SAM,CTR,CFM,CTX,GEN(14)	*Pap,sfa*	+
15	*358 M*	AZT,OFL,AMP,CXM,CAZ,FEP,CIPR,CFZ,SAM,CTR,CFM,CTX,GEN(13)	*Fim,pap,sfa*	−
16	*M-126M*	0	*Fim,pap*	−
17	*1–66 M*	AZT,OFL,AMC,AMP,CXM,SXT,CAZ,FEP,CIPR,CFZ,SAM,CTR,CFM,CTX,GEN(15)	*Fim,pap,sfa*	+
18	*1–807 M*	AMC,AMP,CXM,SXT,FEP,CFZ,SAM,CTR,CFM,CTX(10)	*Fim*	−
19	*425 M*	OFL,AMC,AMP,SXT,CIPR(5)	*Fim,pap,sfa*	+
20	*228 M*	AMP,CFZ,SAM(3)	*Fim*	−
21	*388 M*	AZT,OFL,AMP,CXM,SXT,CAZ,FEP,CIPR,CFZ,SAM,CTR,CFM,CTX,GEN(14)	*Fim,pap,sfa,afa*	+
22	*133 M*	AZT,AMP,CXM,SXT,CAZ,FEP,CFZ,CTR,CFM,CTX(10)	*Fim,pap,sfa*	+
23	*410B*	AMP,CXM,SXT,CFZ,CTR,CFM,CTX,GEN(8)	*Fim*	−
24	*282 M*	0	*Fim,pap,sfa*	−
25	*246 M*	0	*Fim,pap,sfa*	−
26	*199 M*	AMP,SXT,CFZ,SAM(4)	*Fim,pap*	+
27	*333 M* (host)	AZT,OFL,AMP,SXT,CXM,FEP,CIPR,CFZ,SAM,CTR,CFM,CTX,GEN(13)	*Fim,pap,sfa*	+
28	*4SA*	AZT,AMP,SXT,CAZ,CFZ,CTR,CTX,CFM,CXM(9)	*Fim,pap*	−
29	*1–978 M*	AMC,AMP,CXM,SXT,CFZ,CFM(6)	*Fim,pap,sfa*	+
30	*243 M*	0	*Fim,pap,sfa*	+
31	*155 M*	AZT,OFL,AMC,AMP,CXM,CAZ,CIPR,CFZ,SAM,CTR,CFM,CTX(12)	*Fim*	−
32	*142 M*	AZT,OFL,AMP,CXM,SXT,CAZ,FEP,CIPR,CFZ,CTR,CFM,CTX,GEN(13)	*Fim,pap,sfa*	−
33	*3SA*	AZT,OFL,AMC,AMP,CXM,SXT,CAZ,FEP,CIPR,CFZ,SAM,CTR,CFM,CTX,GEN(15)	*Fim,pap,sfa*	+
34	*303 M*	OFL,AMP,CXM,SXT,FEP,CIPR,CFZ,SAM,CTR,CFM,CTX,GEN(12)	*Fim,pap,sfa*	+
35	*305 M*	AMP,GEN(2)	*Fim,pap,sfa*	−
36	*206 M*	OFL,AMP,SXT,CIPR,CFZ,SAM,GEN(7)	*Fim,pap,sfa,afa*	+
37	*8SA*	AZT,OFL,AMI,AMC,AMP,CXM,SXT,CAZ,FEP,CIPR,CFZ,CTR,CFM,CTX(14)	*Fim*	−
38	*173 M*	OFL,AMP,CXM,CIPR,CFZ,CTR,CFM,CTX(8)	*Fim,pap*	+
39	*183 M*	AZT,OFL,AMP,CXM,SXT,FEP,CIPR,CFZ,CTR,CFM,CTX(11)	*Fim,pap,sfa*	+
40	*360 M*	AZT,OFL,AMP,CXM,SXT,CAZ,FEP,CIPR,CFZ,CTR,CFM,CTX(12)	*Fim,pap,sfa*	+
41	*355B*	AZT,OFL,AMP,CXM,SXT,CAZ,FEP,CIPR,CFZ,SAM,CTR,CFM,CTX,GEN(14)	*Fim,pap,sfa*	−
42	*323B*	AZT,OFL,AMC.AMP,CXM,SXT,CAZ,CIPR,CFZ,SAM,CTR,CFM,CTX,GEN(14)	*Fim,pap,sfa*	−
43	*352B*	AMP,SAM(2)	*Pap,afa*	+
44	*461B*	OFL,AMP,CIPR(3)	*Fim,pap,sfa,afa*	+
45	*193 M*	OFL,AMP,SXT,CIPR(4)	*Fim,pap,sfa*	+
46	*121B*	AMP,SXT(2)	*Fim,pap*	+
47	*297B*	AMP,SXT(2)	*Fim,pap,sfa*	+
48	*371B*	AZT,OFL,AMC,AMP,CXM,SXT,CAZ,FEP,CIPR,CFZ,SAM,CTR,CFM,CTX,GEN(15)	*Fim,pap*	−
49	*362B*	OFL,AMI,AMC,AMP,CXM,SXT,FEP,CIPR,CFZ,SAM,CTR,CFM,CTX(13)	*Fim,pap,sfa*	+
50	*393B*	AZT,OFL,AMI,AMP,CXM,SXT,CAZ,FEP,CIPR,CFZ,SAM,CTR,CFM,CTX,GEN(15)	*Fim,pap,sfa*	+
51	*160 M*	AMP(1)	*Fim*	+
52	*383B*	AZT,OFL,AMC,AMP,CXM,SXT,CAZ,FEP,CIPR,CFZ,SAM,CTR,CFM,CTX,GEN(15)	*Fim,pap,sfa*	+

_+_symbolizes sensitivity to the phage; ^−^resistance to the phage.

AZT = Aztreonam, OFL = Ofloxacin, AMI = Amikacin, AMC = Amoxicillin/clavulanic acid, AMP = Ampicillin, CXM = Cefuroxime, SXT=Trimethoprim/sulfamethoxazole, CAZ = Ceftazidime, FEP = Cefepime, CIPR = Ciprofloxacin, CFZ = Cefazolin, SAM = Ampicillin/sulbactam, CTR = Ceftriaxone, CFM = Cefxime, CTX = Cefotaxime, IMI = Imipenem, GEN = Gentamicin.

Bacteriophages are usually highly specific in a sense that most of them can only infect a single species of bacteria^14^. VB_EcoS-Golestan showed no lytic activity against bacteria, including *Acinetobacter baumannii* ATCC 19606*, Staphylococcus aureus* ATCC 43300, *Pseudomonas aeruginosa* ATCC 27853, *Pseudomonas aeruginosa* ATCC 47085, *Staphylococcus epidermidis* PTCC 1435: (*Staphylococcus epidermidis* ATCC 14990)*, Klebsiella pneumoniae* ATCC 700603*, Klebsiella oxytoca* PTCC 1402*: (Klebsiella oxytoca* ATCC 8724), *Staphylococcus saprophyticus* ATCC 15305. However, the phage was active against *Escherichia coli* ATCC 25922. Moreover, the spot test showed that VB_EcoS-Golestan was effective against 28 (53.8%) of the 52 clinical *E. coli* isolates (Table 1). This observation signifies that the phage has a high specificity towards its host in which it was not effective against about 46% of the different isolates of bacterial host (*E. coli*). This feature is a limiting factor for application of the phage in treatment of different *E. coli* infections. This issue can be tackled by using phage cocktails^24^.

Out of a total of 41 MDR isolates, 23 isolates (56%) were sensitive to the lytic activity of the phage. This shows that for phage therapy more than a single phage should be used. Out of the 52 *E. coli* isolates, 25 were resistant to ≥10 antibiotics, of which 15 (60%) isolates were sensitive to the phage. Furthermore, six isolates were resistant to all antibiotics except for amikacin and imipenem, of which 4 isolates (67%) were susceptible to the phage activity (Table 1). This broad resistance to different antibiotics highlights the need for new approaches such as phage therapy. This phage could effectively be applied against both antibiotic sensitive or resistant UPEC isolates causing problems in the treatment of the clinical cases^25^.

### Adhesion fimbria and sensitivity to the phage VB_EcoS-Golestan

The adhesive system and its corresponding genes in UPECs are some of the most common virulence factors of *E. coli* strains in UTI. These virulence factors play an important role in the colonization and invasion of the bladder by UPECs through mediating attachment of the bacteria to the bladder cells surface^5^. The frequencies of the studied virulence genes alone or in combination are presented in Supplementary Table S2. The sequences of *fimH*, *pap*, *sfa*, and *afa* adhesion factors detected in the isolates are deposited in the gene bank with accession numbers of MG041766, LC373009, LC373010, and LC373216, respectively. The most common virulence gene which was detected in all of the UTI isolates was the *fimH* gene. The next frequent virulence genes were *pap* and *sfa* presented in 78.8% (41 out of 52), and 69.2% (36 out of 52) of the isolates, respectively. Finally, *afa* was the least frequent virulence gene found in only 4% (4 out of 52) of the isolates. All isolates harbored the adhesive genes either singly or in combination. Thirty-four isolates (65.3%) were positive for *fimH*, *pap* and *sfa* genes together. Only, three of the isolates (5.7%) were positive for all four genes.

It has been shown that bacterial cell surface structures can serve as receptors for bacteriophages^26^. These structures can be classified according to their structural characteristics, and have several roles, including acting as virulence factors^26,27^. Detection of these receptors by bacteriophage determines the specificity of a phage and its host range. In UPEC isolates, virulence factors such as fimbriae are a good receptors for binding of bacteriophage having tail fibers^26^. Among the isolated *E. coli*, 25 isolates with *pap* gene (61%) were sensitive to the VB_EcoS-Golestan phage. Of the 36 isolates harboring *sfa* gene, 23 isolates (63.9%) were found susceptible to the phage. Moreover, all isolates harboring *afa* gene (4 isolates) were sensitive to the lytic activity of the phage. Furthermore, the 3 isolates which were encoding all of the adhesion genes (*pap*, *sfa*, *fimH*, and *afa*) were also found susceptible to VB_EcoS-Golestan phage (Table 1). These results indicate that there is a significant correlation between virulence factors and bacterial sensitivity to phage (P < 0.05). In other words, presence of these adhesions proteins on the surface of *E. coli* isolates can increase the chance of phage attachment to the host bacterium. Therefore, *E. coli* strains with modifications or lower expression of such receptors might be resistant to bacteriophage infection^26^.

### Stability of the VB_EcoS-Golestan phage

Thermal stability of the phage VB_EcoS-Golestan is shown in Supplementary Fig. S2a. The maximum stability was observed from 37 to 45 °C. The activity of the phage decreased by increasing temperature and was fully deactivated at 75 °C after one hour incubation. The phage showed maximum stability at pH values of 7 and 8, in which no significant differences were observed in the phage titers after 1 h and 24 h incubation. The phage was also stable in the pH values from 5 to 10 after 1 h incubation where there was a negligible decrease in the phage titer after 1 h and 24 h incubation compared to the recorded values for pH of 7 and 8. However, A significant reduction was observed in the phage titer at pH values of 3 and 11 after 1 and 24 h incubation, and it was completely deactivated at pH 2 and 12 (**see** Supplementary Fig. S2b). This data indicates that VB_EcoS-Golestan has high stability in a wide range of temperatures and pH conditions, which is advantageous for potential application of this phage in phage therapy at different environmental settings^28^.

### Cationic ions and phage adsorption rate

**Figure 2:**
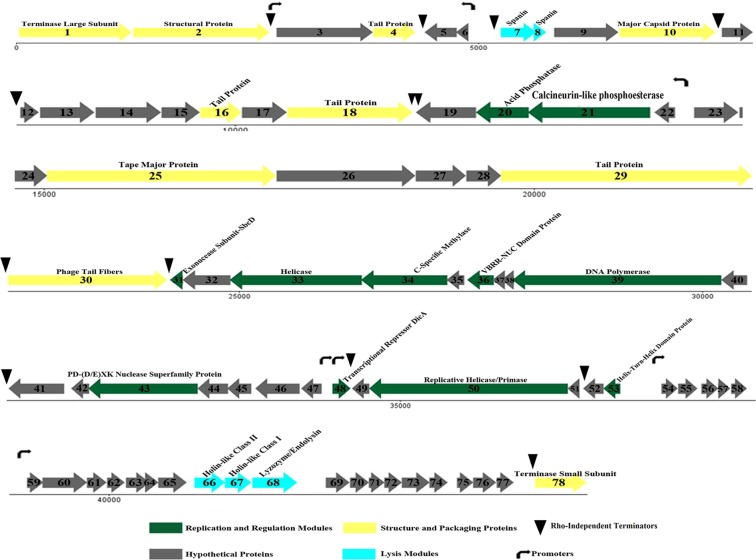
Genomic map of VB_EcoS-Golestan, including the promoter and Rho-independent terminators. Arrows represent 78 ORFs, and the different colors identify the functional categories to which the homologous genes were classified. Gene functions are indicated where they are known. The color codes for gene functions are provided at the bottom of the figure.

Subjecting the phage to 10 mmol/L of Mg^+2^ (MgCl_2_) or Ca^+2^ (CaCl_2_) resulted in a significant increase in the adsorption rate compared to the control (Two-way ANOVA; P < 0.05, Fig. 2). About sixty-six percent (65.8%) of the phage was adsorbed to *Escherichia coli* 333 cells within 5 min in the control mixture (without metal ions). In the cases of adding magnesium chloride or calcium chloride, the adsorption rates were 89.4% and 85.3%, respectively. The highest phage adsorption occurred after 15 minutes, 99.5% in the control and 99.8% in the samples containing cationic ions. Thereafter, no prominent changes were observed. In other words, these cations stabilized the interaction of bacteriophage with its host bacterium. Previous studies reported that cofactors such as Ca^2+^/Mg^2+^ ions can stabilize the fragile interface of the virion with its receptors^28–31^. This means enhanced phage infectivity which can lead to a higher lysis yield in phage therapy.

### One-step growth curve

The latent period and the burst size of VB_EcoS-Golestan phage were determined by one-step growth test. The latent period was approximately 40 min and the burst size was about 100 plaque forming units (pfu) per cell (**see** Supplementary Fig. S3). The burst size of a phage is closely related to its propagation, and having a proper burst size is a desirable characteristic for an effective lytic bacteriophage. Therefore, phages with a short latency period and large burst size have been suggested as proper candidates for phage therapy^14^. Therefore, the observed burst size and relatively short latent period of VB_EcoS-Golestan are desirable characteristics for its potential application in phage therapy.

### *In vitro* lytic activity of the phage

The lytic activity of the phage was examined against *E. coli* 333 culture at its exponential growth phase (OD_600_ = 0.4) with different MOIs of VB_EcoS-Golestan. The highest MOI (MOI 10) resulted in the highest lytic activity within the first hour by an approximately 1.5 Log decrease in the titer of *E. coli* 333 from 10^8^ cfu/ml to about 10^7^ cfu/ml (*P* < 0.0001). After 2 h, using MOIs of 0.1, 1 and 10 let to about 3.5 Log decrease in the bacterium titer to about 10^4^ cfu/ml (*P* < 0.0001). The recorded bacterial titer was almost constant afterward until 8 h after incubation. Using MOI of 0.01 also decreased the titer of the bacterium to the same point (10^4^ cfu/ml) however after 3 h of incubation. Using lower MOIs (0.001 and 0.0001) resulted in moderate decrease of the bacterium titer within early hours (**see** Supplementary Fig. S4a). Therefore, using higher phage concentrations resulted in a faster reduction in the bacterium count which could be due to an increased attachment rate at higher phage titer^32,33^.

Further incubation to 24 h resulted in a significant increase in the bacterium titer in both control and phage treated samples. Even at this point the recorded bacterium titers were about 2 Log less than the control (*P* < 0.0001). The lytic effect of the phage in different MOIs against the host bacterium was also measured by optical absorbance (OD_600_) and the results were consistent with the results of the bacterial cell counts (**see** Supplementary Fig. S4b). In *in vivo* application of a phage, reduction of the bacterial populations to un-infective doses provide more time for the innate immunity to overcome the infection^34,35^. Therefore, determining the optimal titer of a phage is a helpful approach to enhance phage infectivity when faced with its host, especially during the first hours of the treatment^32^. Therefore, the observed decrease in the cell count of the host bacterium over the first three hours of exposure of VB_EcoS-Golestan phage and maintaining such trend up to 8 hours after incubation is a significant feature for the potential application of this phage in phage therapy. However, as demonstrated in Supplementary Fig. S4b, the cell density of the host bacterium increased after 24 h. This increase could be due to the selection of those bacterial strains which phages did not adsorb to them, which then resulted in the overpopulation of the resistant phenotype or emergence of mutated strains in the population of the host bacterium^14,27^.Whatever the reason, this is a critical issue, thus it is vital to tackle this issue. An effective strategy against this issue is using phage cocktail which can effectively control the host bacteria and inhibit possible emergence of phage resistance phenotypes^25,34–37^. Using combination therapy, i.e. simultaneous use of antibiotics and bacteriophages is another solution to overcome such issue^25,38^.

### Restriction profile

EcoRI, EcoRV, NdeI, PstI, BamHI, and HindIII digested the phage genome (**see** Supplementary Fig. S5). The restriction profiles were studied using Sequenti X Gel Analyzer software^39^. This analysis indicated that the phage was a dsDNA virus with a genome size of approximately 45 kDa.

### Genomic analysis

The complete genome of VB_EcoS-Golestan revealed that it does not harbor any harmful gene such as those genes associated with antibiotic resistance, lysogenic, toxins or other virulence factors. This suggests that VB_EcoS-Golestan phage can be introduced as a virulent phage against *E. coli*.

The genome of phage VB_EcoS-Golestan is 44829 bp in length with a G + C content of 50.6%, which is similar to the majority of available genome sequences of *E.coli* with GC contents ranging from 50 to 52%. The genome consisted of 78 open reading frames (ORFs) (Supplementary Table S3) with most of them located on the plus strand (64.1%, 50 ORF). All ORFs begin with ATG codon except for ORFs 24, 29, 68 and 99, which start with TTG. Three types of codons were used for prediction of the ORFs, including TAG as the most common stop codons (50%, 39 ORF) followed with TAA (40%, 31 ORF) and TAG (10%, 8 ORF) (Supplementary Table S3). Using tRNA-Scan and GtRNAdp no tRNA was detected in the genome. Furthermore, seven transcriptional promoter sequences were identified by PHIRE software (Supplementary Table S4). Thirteen Rho-factor-independent terminators were detected in the genome of VB_EcoS-Golestan using ARnold (Supplementary Table S5**)**, which were assessed according to their location, presence of a U-rich tail, and strongly predicted stem-loop secondary structure (ΔG ≤ −10 kcal mol^−1^) as calculated by MFold^31^.

**Figure 3:**
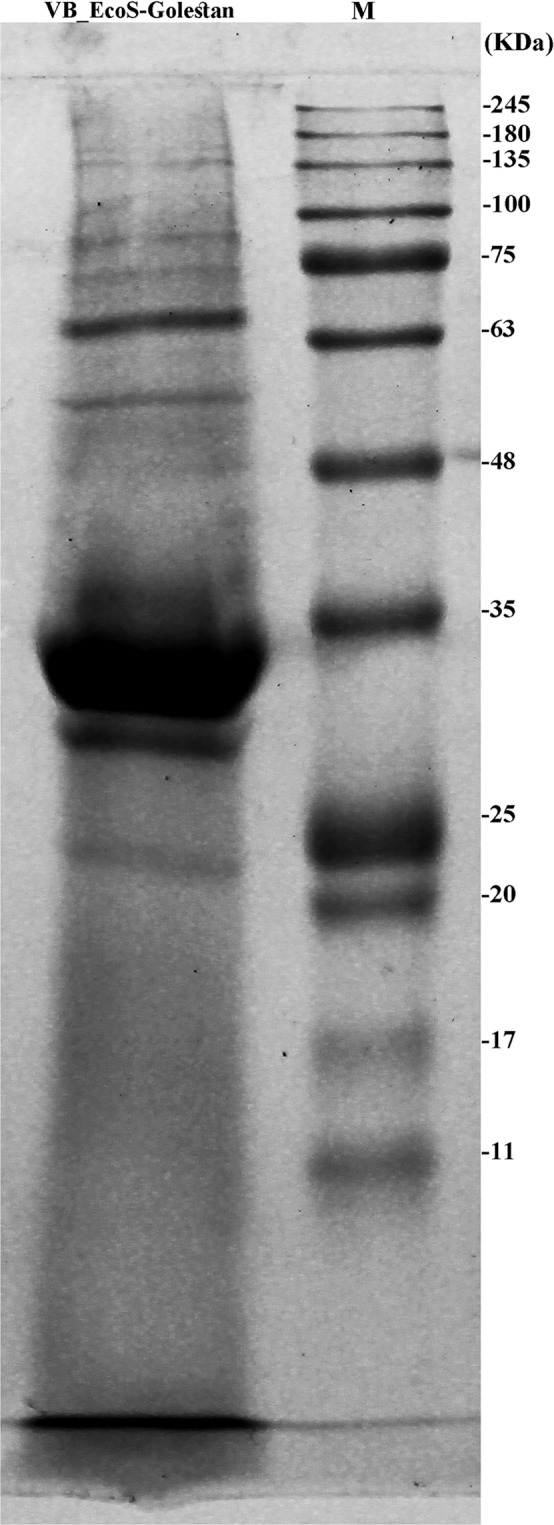
The SDS-PAGE analysis of phage VB_EcoS-Golestan structural proteins on 10% gel staining with Coomassie brilliant blue. M, a standard marker of molecular weight (kDa).

Of the 78 ORFs, 26 were similar to that of the GenBank functional genes. Forty-six were similar to hypothetical proteins with unknown function. The other six had no similarity with any protein available in the databank (NCBI), thus were unique ORFs in this phage and were registered as hypothetical proteins in the GenBank. The VB_EcoS-Golestan genome was organized in separate functional modules containing genes involved in structural and packaging (10 ORFs), replication and regulation (11 ORFs) and cell lysis (5 ORFs) (Fig. 3).

### Structural and packaging proteins

All of the genes encoding structural and packaging proteins are located on the plus strand, including terminase proteins (ORFs 1 and 78), tail proteins (ORFs 4, 16, 18 and 29), tail fiber protein (ORF30), major capsid protein (ORF 10), structural protein (ORF 2) and tape measure protein (ORF 25).

**Figure 4:**
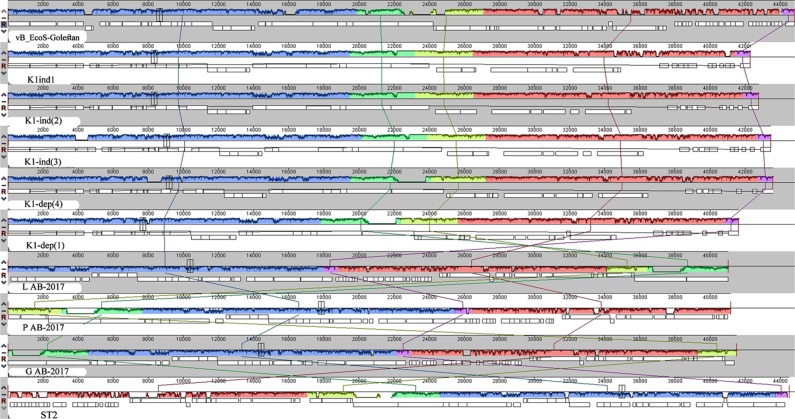
Mauve alignment of the annotated complete genomes of *Escherichia* phage VB_EcoS-Golestan with Escherichia phages ST2, P AB-2017, L AB-2017, K1-dep(1), K1-dep(4), K1-ind(3), K1-ind(2) and K1ind1 (from bottom to top). Genome similarity is represented by a similarity plot within the colored blocks with the height of the plot proportional to the average nucleotide identity. The white regions represent fragments that were not aligned or contained sequence elements specific to a particular genome.

Eleven protein bands, representing virion structural components, were seen in a SDS polyacrylamide Coomassie-stained gel with sizes ranging from 25 to 150 kDa (Fig. 4). A predominant polypeptide band of about 35 kDa is suggestive of major capsid protein as a result of the high capsid protein copy number. The detected molecular mass corresponds to the predicted molecular weight of this protein. Blastp analysis demonstrated that the VB_EcoS-Golestan major capsid protein has resemblance to the *Escherichia* phages ST2, K1-dep(4), K1-dep(3), K1-ind(3), K1-ind(2) and K1-ind(1) (sequence identity ranging from 98% to 96%) major capsid proteins within the *Kagunavirus* genus, *Guernseyvirinae* subfamily, *Siphoviridae* family, according to the ICTV classification of phages.

The DNA packaging system of the tailed phages contains a heterodimeric terminase constituted of large and small subunits where the small subunit is accountable for DNA binding and the large subunit (terminase) that intercedes the prohead is responsible for binding and cleavage of the phage concatameric DNA into single genome units^31^. In VB_EcoS-Golestan the products of ORF1 and ORF78 were predicted as large terminase and small terminase proteins, respectively. These proteins have 96% and 99% identity to the large and small terminase proteins of *Escherichia* phage ST2, respectively. Four ORFs (4, 16, 16 and 29) were predicted as tail proteins, with amino acid sequence identities with orthologous genes of *Escherichia* phages within the *Kagunavirus* genus, ranging from 80% to 99%.

Tail fibers in the phage tail, play a very important role in the initiation of the phage coupling with its bacterial receptors and thus have a role in the host specificity^40^. The tail fiber protein encoded by ORF30 had 88% sequence identity with the tail fiber protein of *Escherichia* phage LM33-P1. ORF 25 of VB_EcoS-Golestan genome encoding a tape measure protein (TMP), the genome second largest gene, contains a HI15114 area in the N-terminal end. It, as a multifunctional protein, has roles in determining the length of the tail (in colaboration with assembly chaperones of the tail), link of the capsid and distal regions of the tail, and genome delivery^40,41^. ORFs 3 and 27 were predicted as hypothetical proteins, while the putative conserved domains of these ORFs were involved in the assembly of the bacteriophage. ORF27 contained a DUF1833 (pfam08875) domain which was predicted as tail assembly chaperone involved in tail assembly. As described above, this ORF in concert with ORF25 (which encodes a tape measure protein) are responsible for determination of the tail length. ORF3 contained a phage SPP1 domain (TIGR01641) and a phage-Mu-F (pfam04233) region toward its C-terminus. These domains are involved in the viral head morphogenesis of double-stranded DNA bacteriophages.

### Replication and regulation proteins

Eleven genes in the vB_EcoS-Golestan genome were predicted to play a role in replication and regulation. ORF20 encodes an acid phosphatase consisting of a HAD-PNKP-C (cd07502) family domain. This family consists of the C-terminal domain of the bifunctional enzyme T4 polynucleotide kinase/phosphatase (PNKP). The PNKP phosphatase domain is able to catalyze hydrolytic removal of the 3′-phosphoryl of RNA, DNA, and deoxynucleoside 3′-monophosphates^42^. ORF31, which encodes an exonuclease subunit SbcD, is comprising of PRK10966 domain and DFU4140 (pfam13600) N-terminal domain. It showed 100% identity with exonuclease subunit SbcD of *Escherichia* phage P AB-2017. The helicase and replicative helicase/ primase encoded by ORFs 33 and 50, demonstrated the highest homology with G AB-2017 ORFs 66 and 52 (98% identity), respectively, which are engaged in replication, recombination, and repair of the phage^43^. The product of ORF39 was predicted as DNA polymerase containing DNA-pol-A superfamily domain with 94% similarity to *Escherichia* phages K1-ind(3), G AB-2017, K1-dep(1) and K1-dep(4). The VRR-NUC protein that is encoded by ORF36 is associated with PD-(D/E)XK nuclease superfamily protein (ORF43), which include restriction modification enzymes. ORFs 48 and 53 encode helix-turn-helix–family DNA binding proteins and are engaged in DNA replication regulation, transcription, telomere maintenance and repair,. Helix-turn-helix proteins are involved in specific identification of the genome of the virus for the beginning of DNA packaging during virus assembly^44^.

### Cell wall lysis proteins

The dsDNA phages of eubacteria use endolysin or muralytic enzymes together with holin, a small membrane protein, to degrade the bacterial cell wall peptidoglycan^45^. In the VB_EcoS-Golestan, genes recognized playing a role in host cell wall lysis included of putative holin class II (ORF66) and I (ORF67) located at the upstream of putative endolysin (ORF68) that contains autolysin (cd00737), muramidase (COG3772) and phage lysozyme (pfam00959) domains. Another protein that contributes to cell lysis of gram-negative bacteria is spanin. This protein is engaged in the outer membrane interuption and also catalyzes the outer and inner membranes fusion in the gram-negative bacteria^45^. In the VB_EcoS-Golestan, spanin protein is encoded by ORF7 and 8. All of the ORFs encoding proteins involving in cell wall lysis of the VB_EcoS-Golestan share high homology to *Escherichia* phages K1ind(2), K1ind(3), K1ind(1), L AB-2017, K1-dep(1), K1-dep(4), G AB-2017, P AB-2017 and ST2 (identity ranging from 90% to 99%).

### The relationship between VB_EcoS-Golestan with other bacteriophages

**Figure 5:**
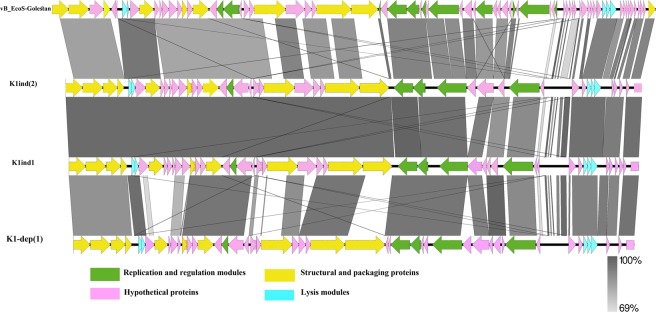
The comparison of the whole genome sequence of the VB_EcoS-Golestan with closet similar phages (*Escherichia* Phages K1ind(2), K1ind2 and K1-dep(1) using Easy fig. The colored arrows indicate ORFs according to their predicted function. The homologous regions between phages are indicated by gray shading.

**Figure 6:**
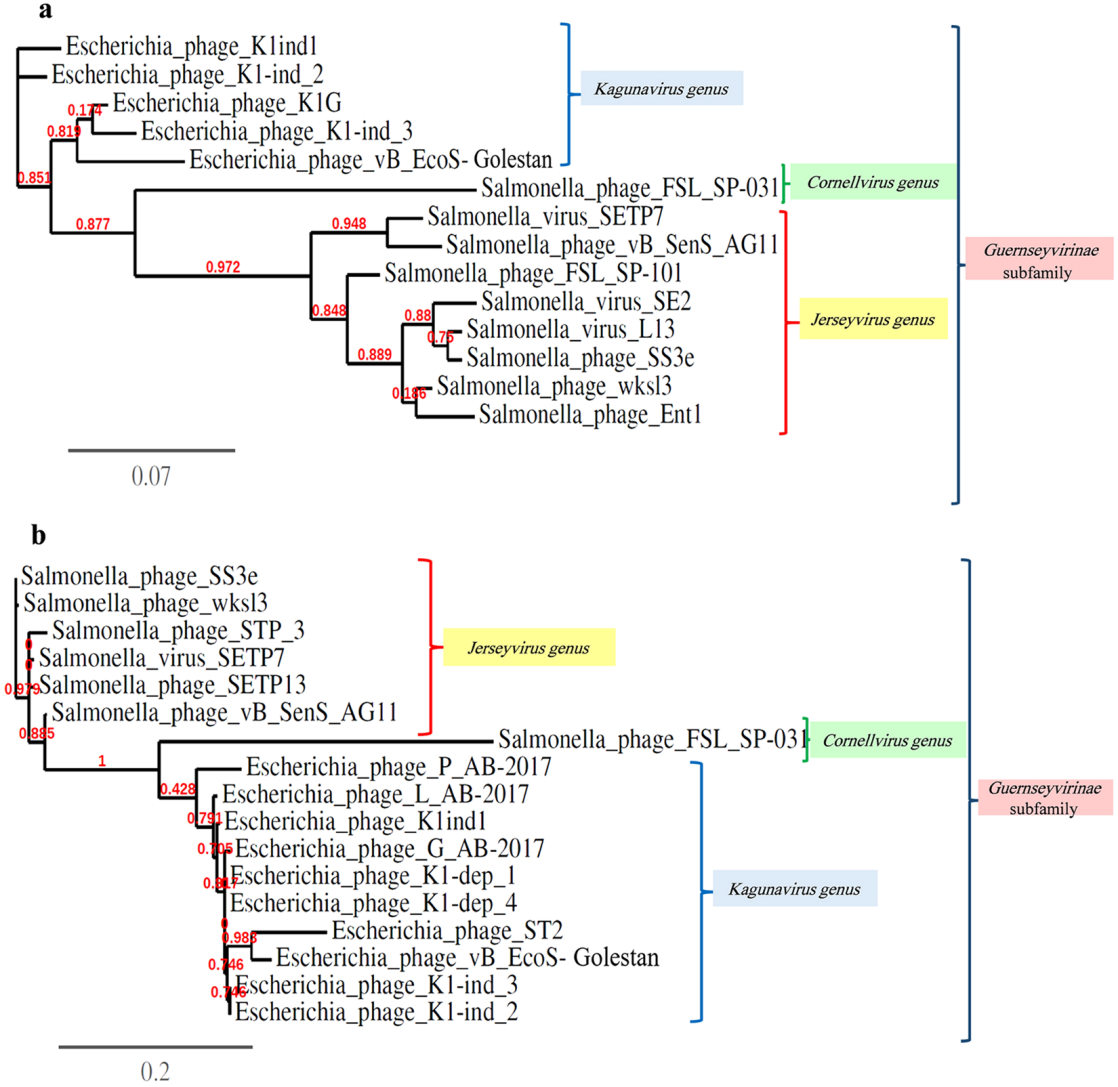
Phylogenetic analysis of the (**a**) major capsid protein, (**b**) DNA polymerase proteins of *Escherichia* phage VB_EcoS-Golestan with phages belonging to the *Guernseyvirinae* subfamily constructed using “One Click” at phylogeny.fr.

Megablast full genome sequence analysis of VB_EcoS-Golestan to identify highly similar sequences with other phages revealed significant similarity (88% identity) to complete sequences of the *Escherichia* phages K1ind(2) (accession no. GU196280.1), K1ind(3) (GU196281.1), K1ind(1) (GU196279.1), P AB-2017 (KY295898.1), L AB-2017 (KY295896.1), K1-dep(1) (GU196278.1), K1-dep(4) (GU196277.1), G AB-2017 (KY295895.1) and ST2 (MF153391.1). All of these bacteriophages belong to the *Kagunavirus* genus, *Guernseyvirinae* subfamily, *Siphoviridae* family, according to ICTV classification of phages. Progressive multiple genome alignments were calculated using Mauve (Fig. 5) and Easy fig (Fig. 6) software to determine the relatedness of VB_EcoS-Golestan genome with the homolog phage (mentioned above) and show a considerable relation between VB_EcoS-Golestan and other *Escherichia* phages within *Kagunavirus* genus.

**Figure 7:**
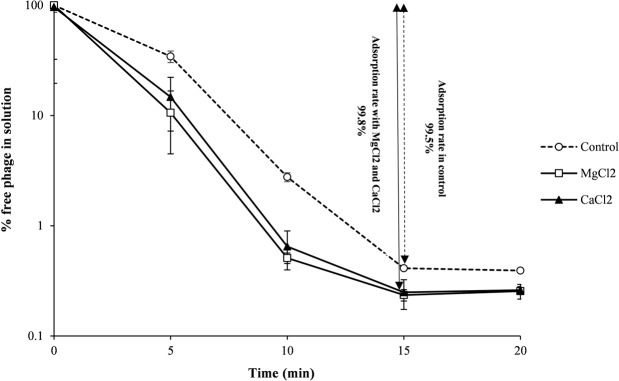
The rate of adsorption of VB_EcoS-Golestan phage to *E. coli* 333 after treatment with CaCl_2_ and MgCl_2._.

To determine the exact taxonomic position of the phage, major capsid and DNA polymerase proteins of VB_EcoS-Golestan with related phages were analyzed using “One Click” of phylogeny.fr server (Fig. 7). The results of the phylogenetic tree confirm the high homology of phage VB_EcoS-Golestan with *Escherichia* phages in *Kagunavirus* genus. Considering the obtained results, it is likely that VB_EcoS-Golestan is a new species within the *Kagunavirus* genus.

## Conclusion

VB_EcoS-Golestan is a virulent phage that belongs to *Kagunavirus* genus of *Guernseyvirinae* subfamily, *Siphoviridae* family. This lytic bacteriophage had a broad host range specificity against both antibiotic sensitive and multidrug-resistant UPEC isolates, a rapid adsorption time, and large burst size, and high stability at a wide range of pH and temperatures, which makes it a promising agent against *E. coli* infections. Moreover, annotation of its whole genome sequence confirmed that there is no virulence factor in its genome including, toxin, lysogenic or antibiotic resistance genes. Therefore, VB_EcoS-Golestan is a potential agent for phage therapy of UTI caused by *E. coli*.

## Material and methods

### Bacterial isolation

Fifty-two *E. coli*, isolated from UTI with colony count of ≥10^5^ CFU/ml, were collected from the hospitals located in the city of Gorgan, Golestan province, Iran. This study was approved by the local ethics committee (Golestan University of Medical Sciences) (IR.GOMS.REC.1394.209). Informed consent was obtained from all participants and/or their legal guardians. All samplings were performed in compliance with relevant laws and institutional guidelines and in accordance with the ethical standards of the Helsinki Declaration. All of the *E. coli* isolated were subjected to biochemical characterization following Mahon *et al*.^46^. All isolates were cultured in brain heart infusion (BHI) broth and stored at −70 °C until further use.

### Antibiotic sensitivity of clinical isolates

Seventeen antibiotics were employed to determine the antibiotic susceptibility profile of the 52 *E. coli* isolates using disk diffusion method on Muller Hinton agar according to Clinical and Laboratory Standards Institute (CLSI) protocols^47^. The following antibiotics (Rosco, Denmark) were included: Aztreonam (30 µg), Ofloxacin (5 µg), Amikacin (30 µg), Amoxicillin/clavulanic acid (20/10 µg), Ampicillin (10 µg), Cefuroxime (30 µg), Trimethoprim/sulfamethoxazole (25 µg), Ceftazidime (30 µg), Cefepime (30 µg), Ciprofloxacin (5 µg), Cefazolin (30 µg), Ampicillin/sulbactam (10/10 µg), Ceftriaxone (30 µg), Cefxime (5 µg), Cefotaxime (30 µg), Imipenem (10 µg), Gentamicin (10 µg).

### Detection of virulence factors in the bacterial isolates

Bacterial DNA was isolated using the phenol-chloroform method. The presence of virulence factors *fimH*, *pap*, *sfa*, and *afa* in *E. coli* isolates, was detected by PCR using specific primer sets designed for these adhesion genes, as described previously^48^. The PCR product of any positive sample was then sequenced by sanger sequencing (Macrogen company, South Korea) and then identified by BLAST alignment tool and deposited in GenBank. The results were used to identify any correlation between the presence of virulence factors and observed sensitivity to the phage.

### Phage isolation

Municipal wastewater samples were collected from city of Gorgan, Golestan province, Iran. Twenty ml of the supernatant from centrifuged (12,000 × g, 10 min) wastewater was mixed with 20 ml 2X BHI broth containing the *E. coli* isolates (in exponential phase, OD_600_ = 1). After 24 h incubation at 37 °C, the suspension was centrifuged and the supernatant was filtrated using a sterile syringe filter with a 0.22 µm pore size (Gilson, UK). To determine the phage presence, 10 µl of the filtrate and the *E. coli* isolate (100 µl) were mixed with a 0.7% melted top soft agar and poured on a plate of brain heart infusion agar. Plaques were identified after overnight incubation at 37 °C. A single plaque on the bacterial lawn was pulled out and mixed with 20 ml of the isolated *E. coli* suspension, then incubated at 37 °C for 18 h. Then, the double-layer plaque assay was carried out. This was repeated for three times in order to obtain a pure stock of the isolated phage^29^. Multidrug-resistant *E. coli* isolate 333 was used as a host for the phage isolation.

### Phage purification

The phage suspension (~ 10^10^ pfu/ml) was centrifuged for 15 min at 13,000×g and the supernatant was filtered (0.22 µm, Gilson, UK), then DNase (1 µg/µl) and RNase (1 µg/µl) were added to the filtrate (1 h at 37 °C) to remove any bacterial DNAs and RNAs. NaCl and polyethylene glycol (PEG) 8000 were added in the phage suspension at final concentrations of 1 M and 10%, respectively. The mixture was stored at 4 °C overnight. The phage was precipitated by 30 min centrifugation at 13,000×g at 4 °C. Two ml of SM buffer (2% gelatin, 5 ml; 1 M Tris-Cl pH 7.5, 50 ml; MgSO4_7H2O, 2 g; NaCl, 5.8 g and ddH2O to 1,000 ml) was used to re-suspend the pellet. The concentrated phage was loaded on a glycerol step gradient (SM buffer with 40 and 5 percent glycerol) and subjected to 2 h ultracentrifugation at 150,000 × g and at 4 °C (Backman L5–65 ultracentrifuge, SW28 rotor). The pellet was re-suspended with SM buffer and stored at 4 °C until further use^43,49^.

### Determination of the host range

The lytic activity of the isolated phage was examined against the 52 clinical isolates of *E. coli* using standard spot assay (Table 1). Briefly, 10 µl of the purified phage was spotted in the center of double agar overlay culture of each isolate and incubated at 37 °C. After overnight incubation, plate with clear lytic zone was considered as susceptible to phage-mediated lysis^14^. Several gram-negative and positive standard bacteria (obtained from the Department of Microbiology, Golestan University of Medical Sciences, Iran) were also used to investigate the phage host range.

### Electron microscopy

A drop of the purified phage (~10^12^pfu/ml) was spotted on a carbon-coated copper grid. Then, 10 µl of uranyl acetate 2% was added to the surface of the grid for 30 s, then the excess was removed by filter paper^50^. The fixed sample was studied using Fei Philips TEM, CM-10 (Japan).

### Phage stability

Thermal and pH stability tests were conducted as previously described^32^. For thermal stability test, the phage suspensions were incubated at 37 (Control), 45, 50, 55, 60, 70 and 75 °C for 1 h, and its pH stability was studied at pH values from 2 to 12. For this, 100 microliters of the phage suspensions were added to 900 µl SM buffer at the desired temperature, incubated for 1 h and 24 h, then the phage titer was evaluated using soft agar overlay assay^32^. The same was done to study its pH stability, however at the constant temperature of 37 °C.

### Analysis of calcium and magnesium ions effects on adsorption rate

To evaluate the effects of cationic ions on the phage, MgCl_2,_ and CaCl_2_ (each at a final concentration of 10 mM) were added to the phage infected cultures. Samples were collected at 0, 5, 10, 15 and 20 min intervals to determine the unabsorbed phage titer and reported as a percentage of the initial phage count^28,32^.

### One-step growth

Latent period and phage burst size were determined by one-step growth test following Li *et al*. (2014) with some modifications. In brief, *E. coli* cells (*E. coli* 333 isolate) were pelleted by centrifugation and re-suspended in fresh BHI broth (2 ml) (~10^9^ cfu/ml). The phage was added at a multiplicity of infection (MOI) of 0.01 and allowed to be adsorbed for 15–20 min at 37 °C, then centrifuged at 13,000×g for 1 min. Twenty milliliters of BHI broth was used to re-suspend the pellet and then incubated at 37 °C. Samples (100 µl) were collected up to 120 min at 10 min intervals and then tittered using the soft agar overlay plate^30^.

### Bacteriolytic characteristic of the phage

The bacteriolytic activity of the phage in different MOIs was determined using a modified version of our previous study^32^. Ten milliliters of BHI broth was impregnated with 300 microliters of the overnight host culture and incubated at 37 °C until reaching to the optimal density of 0.4 (early logarithmic phase). Different MOIs of 0.0001, 0.001, 0.01, 0.1, 1 and 10 of the phage were used to inoculate the bacterial culture and incubated at 37 °C. Samples were collected at 1, 2, 3, 4, 5, 6, 7, 8 and 24 h and measured using optical densitometry (Eppendorf Bio Photometer plus, Germany) at 600 nm. Moreover, 100 µl of each taken sample was diluted and cultured on 2% blood agar to quantify the bacterium titer (cell count)^32^.

### DNA genome extraction and restriction analyses

The genomic DNA was extracted from purified phage that was subjected to DNase (1 µg/µl) and RNase (1 µg/µl) (Thermo Fisher Scientific, Germany) using PureLink Viral DNA mini kit (Thermo Scientific Fisher, Germany) according to the manufacturer’s protocol. Restriction enzymes including EcoRV, NdeI, HindIII, BamHI, EcoRI, and PstI were used to digest the DNA following the manufacturer’s instruction (Thermo Fisher Scientific, Germany) and assayed by electrophorese on 0.8% agarose gel.

### Proteomics analysis of the phage

To determine the proteomic profile of the phage, the ultracentrifuged phage particles were subjected to SDS polyacrylamide gel electrophoresis (SDS-page) analysis. Loading buffer (0.001% (w/v) bromophenol blue, 50 mM Tris-HCl (pH 6.8), 5% (v/v) 2-mercaptoethanol, 10% (w/v) glycerol and 2% (w/v) SDS) was mixed with 21 milliliters of the phage suspension and boiled for 10 min. The phage structural proteins separated on 10% acrylamide gel and visualized with Coomassie brilliant blue R250. Pre-stained protein ladder sizing from 18 to 240 kDa (Cinacolon, Iran) was used as the standard PAGE ruler^43,51^.

### Whole genome sequence

The bacteriophage DNA was sequenced using Illumina Hiseq. 2005 high throughput sequencing (Macrogen Company, South Korea). The complete genome sequence of the phage was assembled using denovo genome assembler with an average of 999-fold coverage. The open reading frames (ORFs) were identified by GeneMarks (http://exon.gatech.edu/GeneMark/heuristic_gmhmmp.cgi)^52^ and PHAST (http://phast.wishartlab.com/)^53^. The ORFs functions were annotated using protein basic local alignment search tool (Blastp) of NCBI server (http: //www.ncbi.nlm.nih. 160 gov/blast/)^54^. A physical map of the annotated phage genome was generated using DNA plotter^55^. The isoelectric pH and molecular weight of the ORFs were identified using Expasy compute PI/MV tools (http://web.expasy.org/compute_pi)^56^. Putative tRNAs were predicted using tRNA Scan-SE (http://lowelab.ucsc.edu/tRNAscan-SE)^57^ and GtRNAdb (http://gtrnadb.ucsc.edu)^58^. The conserved regulatory elements, such as phage-specific promoters and Rho-factor independent terminators were searched by PHIRE^59^ and ARNOLD servers (http://rna.igmors.upsud. fr/toolbox/Arnold/)^60^.

In order to determine the relatedness of the phage genome with the homolog phages, Mauve^61^ and EasyFig.^62^ softwares were used for progressive multiple genome alignment and comparison of the phages nucleic and amino acid sequences with that of the homolog phages sequences available at NCBI database. Phylogenetic analysis of the phage DNA polymerase and major capsid protein was performed using “One click” tool of phylogeny.fr server (http://www.phylogeny.fr/)^63^. The complete genomic sequence of the phage was submitted in the NCBI database under the accession no. MG099933.1.

### Statistical analysis

The experiments were done in triplicate when required, and the mean ± SD was reported. T-test, Two-way ANOVA and One-way ANOVA- Repeated measures were used for statistical analyses using Graph Pad Prism 6.05 software. The comparison of prevalence rates was performed using Pearson Chi-square test with SPSS software 16.0. A *P* ≤ 0.05 was considered as significant.

## Supplementary information

Supplementary information.

## References

[1] Foxman B (2010). The epidemiology of urinary tract infection. *Nature Reviews*. Urology.

[2] Hojati Z, Zamanzad B, Hashemzadeh M, Molaie R, Gholipour A (2015). The FimH gene in uropathogenic *Escherichia coli* strains isolated from patients with urinary tract infection. Jundishapur J. Microbiol..

[3] Qin X (2013). Comparison of adhesin genes and antimicrobial susceptibilities between uropathogenic and intestinal commensal *Escherichia coli* strains. PLoS One..

[4] Wong CKM (2017). Antibiotic resistance rates and physician antibiotic prescription patterns of uncomplicated urinary tract infections in southern Chinese primary care. PLoS One..

[5] Rahdar M, Rashki A, Miri HR, Ghalehnoo MR (2015). Detection of pap, sfa, afa, foc, and fim adhesin-encoding operons in uropathogenic *Escherichia coli* isolates collected from patients with urinary tract infection. Jundishapur J. Microbiol..

[6] Shetty A, Kumar S, Shekar M, Shetty A, Karunasagar I (2014). Prevalence of adhesive genes among uropathogenic *Escherichia coli* strains isolated from patients with urinary tract infection in Mangalore. Indian J. Med. Microbiol..

[7] Chibeu A (2012). Bacteriophages with the ability to degrade uropathogenic *Escherichia coli* biofilms. Viruses..

[8] Wagenlehner FM, Naber KG (2006). Treatment of bacterial urinary tract infections: presence and future. Eur. Urol..

[9] Dehbanipour R, Rastaghi S, Sedighi M, Maleki N, Faghri J (2016). High prevalence of multidrug-resistance uropathogenic *Escherichia coli strains*, Isfahan, Iran. J. Nat. Sci. Biol. Med..

[10] Terlizzi ME, Gribaudo G, Maffei ME (2017). UroPathogenic *Escherichia coli* (UPEC) infections: virulence factors, bladder responses, antibiotic, and non-antibiotic antimicrobial strategies. Front. Microbiol..

[11] Olorunmola FO, Kolawole DO, Lamikanra A (2013). Antibiotic resistance and virulence properties in *Escherichia coli* strains from cases of urinary tract infections. Afr J. Infect. Dis..

[12] Shahrbabak SS (2013). Isolation, characterization and complete genome sequence of PhaxI: a phage of *Escherichia coli* O157: H7. Microbiology..

[13] Hanlon GW (2007). Bacteriophages: an appraisal of their role in the treatment of bacterial infections. Int. J. Antimicrob. Agents.

[14] Amarillas L (2017). Isolation and characterization of phiLLS, a novel phage with potential biocontrol agent against multidrug-resistant *Escherichia coli*. Front. Microbiol..

[15] Sváb D (2018). Identification and characterization of T5-Like bacteriophages representing two novel subgroups from food products. Front. Microbiol..

[16] Ackermann HW (1998). Tailed bacteriophages: the order *caudovirales*. Adv. Virus Res..

[17] Fauquet CM, Mayo MA, Maniloff J, Desselberger U, Ball L (2005). Virus taxonomy. Eighth report of the international committee on taxonomy of viruses..

[18] Ali I, Rafaque Z, Ahmed S, Malik S, Dasti JI (2016). Prevalence of multi-drug resistant uropathogenic *Escherichia coli* in Potohar region of Pakistan. Asian Pac. J. Trop. Biomed..

[19] Ramírez-Castillo FY (2018). An evaluation of multidrug-resistant Escherichia coli isolates in urinary tract infections from Aguascalientes, Mexico: cross-sectional study. Ann. Clin. Microbiol. Antimicrob..

[20] Reis, A. C. C. *et al*. Ciprofloxacin resistance pattern among bacteria isolated from patients with community-acquired urinary tract infection. *Rev. Inst. Med. Trop. Sao Paulo*. **58** (2016). 10.1590/S1678-9946201658053 PMC4964322 27410913

[21] Shams S (2018). Imipenem resistance in clinical *Escherichia coli* from Qom, Iran. BMC. Res. Notes..

[22] Zhang H (2018). Carbapenem susceptibilities of Gram-negative pathogens in intra-abdominal and urinary tract infections: updated report of SMART 2015 in China. BMC. Infect. Dis..

[23] Devrim F (2018). The emerging resistance in nosocomial urinary tract infections: from the pediatrics perspective. Mediterr. J. Hematol. Infect. Dis..

[24] Yu YP (2013). Isolation and characterization of five lytic bacteriophages infecting a Vibrio strain closely related to *Vibrio owensii*. FEMS Microbiol. Lett..

[25] Kalatzis PG, Bastías R, Kokkari C, Katharios P (2016). Isolation and characterization of two lytic bacteriophages, φSt2 and φGrn1; phage therapy application for biological control of *Vibrio alginolyticus* in aquaculture live feeds. PLoS One..

[26] León M, Bastías R (2015). Virulence reduction in bacteriophage resistant bacteria. Front. Microbiol..

[27] Hyman P, Abedon ST (2010). Bacteriophage host range and bacterial resistance. Adv. Appl. Microbiol..

[28] Jamal M, Hussain T, Das CR, Andleeb S (2015). Characterization of *Siphoviridae* phage Z and studying its efficacy against multidrug-resistant *Klebsiella pneumoniae* planktonic cells and biofilm. J. Med. Microbiol..

[29] Yazdi M, Bouzari M, Ghaemi EA (2018). Isolation and characterization of a lytic bacteriophage (vB_PmiS-TH) and its application in combination with ampicillin against planktonic and biofilm forms of *Proteus mirabilis* isolated from urinary tract infection. J. Mol. Microbiol. Biotechnol..

[30] Li L, Zhang Z (2014). Isolation and characterization of a virulent bacteriophage SPW specific for *Staphylococcus aureus* isolated from bovine mastitis of lactating dairy cattle. Mol. Biol. Rep..

[31] Turner D (2017). Characterisation and genome sequence of the lytic *Acinetobacter baumannii* bacteriophage vB_AbaS_Loki. PLoS One..

[32] Yazdi, M., Bouzari, M. & Ghaemi, E. A. Isolation and characterization of a potentially novel *Siphoviridae* phage (vB_SsapS-104) with lytic activity against *Staphylococcus saprophyticus* isolated from urinary tract infection. *Folia Microbiol. (Praha)*, 1–12 (2018). 10.1007/s12223-018-0653-9 30284669

[33] Shen GH (2012). Isolation and characterization of φkm18p, a novel lytic phage with therapeutic potential against extensively drug resistant *Acinetobacter baumannii*. PLoS One..

[34] Hyman, P. Phages for Phage Therapy: Isolation, Characterization, and Host Range Breadth. *Pharmaceuticals*. **12**, 35; 10.3390/ph12010035(2019). 10.3390/ph12010035 PMC6469166 30862020

[35] Leung CYJ, Weitz JS (2017). Modeling the synergistic elimination of bacteria by phage and the innate immune system. J Theor Biol..

[36] Tanji Y (2005). Therapeutic use of phage cocktail for controlling *Escherichia coli* O157: H7 in gastrointestinal tract of mice. J Biosci Bioeng..

[37] Shahin, K., Bouzari, M., Wang, R. & Yazdi, M. Prevalence and molecular characterization of multidrug-resistant Shigella species of food origins and their inactivation by specific lytic bacteriophages. *Int J Food Microbiol*, 10.1016/j.ijfoodmicro.2019.108252 (2019). 10.1016/j.ijfoodmicro.2019.108252 31276953

[38] Oechslin F (2016). Synergistic interaction between phage therapy and antibiotics clears *Pseudomonas aeruginosa* infection in endocarditis and reduces virulence. J Infect Dis..

[39] Shahin K, Bouzari M, Wang R (2018). Isolation, characterization and genomic analysis of a novel lytic bacteriophage vB_SsoS-ISF002 infecting *Shigella sonnei* and *Shigella flexneri*. J. Med. Microbiol..

[40] Li E (2016). Isolation and characterization of a bacteriophage phiEap-2 infecting multidrug resistant *Enterobacter aerogenes*. Sci. Rep..

[41] Abatángelo V (2017). Broad-range lytic bacteriophages that kill *Staphylococcus aureus* local field strains. PLoS One..

[42] Keppetipola N, Shuman S (2006). Mechanism of the phosphatase component of Clostridium thermocellum polynucleotide kinase-phosphatase. RNA..

[43] Yazdi M, Bouzari M, Ghaemi EA (2018). Genomic analyses of a novel bacteriophage (VB_PmiS-Isfahan) within *Siphoviridae* family infecting *Proteus mirabilis*. Genomics..

[44] Greive SJ (2015). DNA recognition for virus assembly through multiple sequence-independent interactions with a helix-turn-helix motif. Nucleic Acids Res..

[45] Young R (2014). Phage lysis: three steps, three choices, one outcome. J. Microbiol..

[46] Mahon, C. R., Lehman, D. C. & Manuselis, G. *Textbook of diagnostic microbiology-E-Book*. (Elsevier Health Sciences, 2014).

[47] Wayne, P. A. Performance standards for antimicrobial susceptibility testing. 28th ed. CLSI supplement M100. *Clinical and Laboratory Standards institute*. (2018).

[48] Yazdi M, Bouzari M, Ghaemi EA (2018). Detection of fim, pap, sfa and afa adhesin-encoding operons in *Escherichia coli* strains isolated from urinary tract infections. Mljgoums..

[49] Sambrook, J., Fritsch, E. F. & Maniatis, T. *Molecular cloning*. **Vol. 2** (Cold spring harbor laboratory press New York, 1989).

[50] Shahin K, Bouzari M (2018). Bacteriophage application for biocontrolling *Shigella flexneri* in contaminated foods. J. Food Sci. Technol..

[51] Ghasemi SM, Bouzari M, Baygloo NS, Chang HI (2014). Insights into new bacteriophages of *Lactococcus garvieae* belonging to the family *Podoviridae*. Arch. Virol..

[52] Besemer J, Borodovsky M (2005). GeneMark: web software for gene finding in prokaryotes, eukaryotes and viruses. Nucleic Acids Res..

[53] Zhou Y, Liang Y, Lynch KH, Dennis JJ, Wishart DS (2011). PHAST: a fast phage search tool. Nucleic Acids Res..

[54] Altschul SF (1997). Gapped BLAST and PSI-BLAST: a new generation of protein database search programs. Nucleic Acids Res..

[55] Carver T, Thomson N, Bleasby A, Berriman M, Parkhill J (2009). DNAPlotter: circular and linear interactive genome visualization. Bioinformatics..

[56] Gasteiger, E. *et al*. *Protein identification and analysis tools on the ExPASy server*. (Springer, 2005). 10.1385/1-59259-584-7:531 10027275

[57] Schattner P, Brooks AN, Lowe TM (2005). The tRNAscan-SE, snoscan and snoGPS web servers for the detection of tRNAs and snoRNAs. Nucleic Acids Res..

[58] Chan PP, Lowe TM (2009). GtRNAdb: a database of transfer RNA genes detected in genomic sequence. Nucleic Acids Res..

[59] Lavigne R, Sun W, Volckaert G (2004). PHIRE, a deterministic approach to reveal regulatory elements in bacteriophage genomes. Bioinformatics..

[60] Naville M, Ghuillot-Gaudeffroy A, Marchais A, Gautheret D (2011). ARNold: a web tool for the prediction of Rho-independent transcription terminators. RNA Biol..

[61] Darling AE, Mau B, Perna N (2010). T. progressiveMauve: multiple genome alignment with gene gain, loss and rearrangement. PLoS One..

[62] Sullivan MJ, Petty NK, Beatson SA (2011). Easyfig: a genome comparison visualizer. Bioinformatics..

[63] Dereeper A (2008). Phylogeny. fr: robust phylogenetic analysis for the non-specialist. Nucleic Acids Res..

