# Supplementary material for: Isolation, Characterization and Genomic Analysis of a Novel Bacteriophage VB_EcoS-Golestan Infecting Multidrug-Resistant Escherichia coli Isolated from Urinary Tract Infection
Source: Sci Rep. 2020 May 6;10:7690. doi: 10.1038/s41598-020-63048-x (PMC7203180; doi:10.1038/s41598-020-63048-x)
Supplement: Supplementary file 1 — Supplementary information. [file 41598_2020_63048_MOESM1_ESM.doc]

**Isolation, Characterization and Genomic Analysis of a Novel Bacteriophage VB_EcoS-Golestan Infecting Multidrug Resistant *Escherichia coli* Isolated from Urinary Tract Infection**

Mahsa Yazdi^1^, Majid Bouzari^1*^, Ezzat Allah Ghaemi^2*^, Khashayar Shahin^1^

1 Department of Biology, Faculty of Sciences, University of Isfahan, 81746-73441 Isfahan, Iran. E. mails: [mahsayazdi92@gmail.com](mailto:mahsayazdi92@gmail.com), [bouzari@sci.ui.ac.ir](mailto:bouzari@sci.ui.ac.ir), biokhashayar@yahoo.com

2 Laboratory Sciences Research Center, Golestan University of Medical Sciences, 4934174515 Gorgan, Iran. E. mail: dr.ghaemi@goums.ac.ir

**Supplementary Table S1.** Antibiotic resistance pattern of 52 isolates of *E. coli* isolated from urinary tract infection.

| **Sensitive** | |  | **Semi sensitive** | |  | **Resistant** | | **Antibiotics** |
| --- | --- | --- | --- | --- | --- | --- | --- | --- |
| % | **Number** |  | **%** | **Number** |  | **%** | **Number** |
| 42.3 | 22 |  | 11.5 | 6 |  | 46.2 | 24 | Aztreonam |
| 31.5 | 20 |  | 0 | 0 |  | 61.5 | 32 | Ofloxacin |
| 88.5 | 46 |  | 3.8 | 2 |  | 7.7 | 4 | Amikacin |
| 42.3 | 22 |  | 30.8 | 16 |  | 26.9 | 14 | Amoxicillin/clavulanic acid |
| 7.7 | 4 |  | 0 | 0 |  | 92.3 | 48 | Ampicillin |
| 19.2 | 10 |  | 21.2 | 11 |  | 59.6 | 31 | Cefuroxime |
| 25 | 13 |  | 1.9 | 1 |  | 73.1 | 38 | Trimethoprim/sulfamethoxazole |
| 50 | 26 |  | 13.5 | 7 |  | 36.5 | 19 | Ceftazidime |
| 48.1 | 25 |  | 7.7 | 4 |  | 44.2 | 23 | Cefepime |
| 38.5 | 20 |  | 0 | 0 |  | 61.5 | 32 | Ciprofloxacin |
| 30.8 | 16 |  | 1.9 | 1 |  | 67.3 | 35 | Cefazolin |
| 34.6 | 18 |  | 13.5 | 7 |  | 51.9 | 27 | Ampicillin/sulbactam |
| 40.4 | 21 |  | 3.8 | 2 |  | 55.8 | 29 | Ceftriaxone |
| 36.5 | 19 |  | 0 | 0 |  | 63.5 | 33 | Cefxime |
| 38.5 | 20 |  | 5.8 | 3 |  | 55.8 | 29 | Cefotaxime |
| 100 | 52 |  | 0 | 0 |  | 0 | 0 | Imipenem |
| 55.8 | 29 |  | 1.9 | 1 |  | 42.3 | 22 | Gentamicin |

**Supplementary Table S2.** Frequency of virulence factor genes in UTI *E. coli* isolates.

| Virulence factors (alone or in combination) | Number of positive strains (%) |
| --- | --- |
| *fimH*  *pap*  *sfa*  *afa*  *fimH, pap*  *fimH, sfa*  *fim*H*, afa*  *pap, sfa*  *pap, afa*  *Sfa,afa*  *pap, fimH, sfa*  *pap, fimH, sfa, afa* | 52 (100)  41 (78.8)  36 (69.2)  4 (7.6)  41 (78.8)  36 (69.2)  4 (7.6)  34 (65.3)  3 (5.7)  3 (5.7)  34 (65.3)  3 (5.7) |

**Supplementary Table S3. Phage VB_EcoS-Golestan gene annotations.**

| **ORF** | **Position (nt)** | | **Strand** | **Codon** | | **Size (aa)^1^** | **E value** | **Identity (%)** | **Closest hit (accession number)** | **Conserved protein domain family** | **Predictive Function** | **pI^3^** | **Mw^2^ (Da)** | **Accession**  **number** |
| --- | --- | --- | --- | --- | --- | --- | --- | --- | --- | --- | --- | --- | --- | --- |
|  | **From** | **To** |  | **Start** | **Stop** |  |  |  |  |  |  |  |  |  |
| 1 | 28 | 1251 | + | ATG | TAA | 407 | 0.0 | 99 | Putative terminase large subunite[Escherichia phage ST2] ASH99401.1 | pfam 03237,TIGR01547 | Putative terminase large subunite | 6.72 | 45839.90 | ST93225.1 |
| 2 | 1264 | 2745 | + | ATG | TAA | 493 | 0.0 | 97 | Putative structural protein [Escherichia phage K1-ind(3)] ADA82446.1 | Pfam13264 | Putative structural protein | 4.93 | 54758.48 | AST93226.1 |
| 3 | 2815 | 3858 | + | ATG | TAA | 347 | 0.0 | 95 | Hypothetical protein [Escherichia phage ST2] ASH99398.1 | TIGR01641, pfam04233 | Hypothetical protein | 5.30 | 38366.38 | AST93227.1 |
| 4 | 3858 | 4319 | + | ATG | TGA | 153 | 2/00E-98 | 95 | Putative tail protein [Escherichia phage L AB-2017] AQN31847.1 |  | Putative tail protein | 4.50 | 16167.38 | AST93228.1 |
| 5 | 4415 | 4759 | - | ATG | TGA | 114 | - | - | - |  | Hypothetical protein | 8.78 | 12136.06 | AST93229.1 |
| 6 | 4756 | 4857 | - | ATG | TGA | 33 | - | - | - |  | Hypothetical protein | 3.43 | 3423.06 | AST93230.1 |
| 7 | 5234 | 5620 | + | ATG | TAG | 128 | 3/00E-86 | 98 | Putative spanin [Escherichia phage K1-dep(1)] YP_009168838.1 |  | Putative spanin | 9.30 | 13875.59 | AST93231.1 |
| 8 | 5586 | 5729 | + | ATG | TAG | 47 | 1/00E-24 | 96 | [Putative spanin [Escherichia phage K1ind1]ADA82351.1](https://blast.ncbi.nlm.nih.gov/Blast.cgi#alnHdr_282547294) |  | Putative spanin | 4.46 | 5391.19 | AST93232.1 |
| 9 | 5812 | 6513 | + | ATG | TAA | 233 | 2/00E-163 | 99 | Hypothetical protein [Escherichia phage ST2] ASH99394.1 |  | Hypothetical protein | 5.84 | 25438.71 | AST93233.1 |
| 10 | 6516 | 7565 | + | ATG | TAA | 349 | 0.0 | 98 | Putative major capsid protein [Escherichia phage ST2] ASH99393.1 |  | Putative major capsid protein | 4.78 | 37894.42 | AST93234.1 |
| 11 | 7627 | 7968 | + | ATG | TAA | 113 | 4/00E-50 | 73 | Hypothetical protein [Escherichia phage K1-dep(4)] YP_009168791.1 |  | Hypothetical protein | 5.61 | 11556.90 | AST93235.1 |
| 12 | 8005 | 8184 | + | ATG | TAA | 59 | 3/00E-33 | 95 | Hypothetical protein [Escherichia phage ST2] ASH99390.1 |  | Hypothetical protein | 5.26 | 6653.30 | AST93236.1 |
| 13 | 8188 | 8700 | + | ATG | TAA | 168 | 2/00E-113 | 97 | Hypothetical protein [Escherichia phage K1-dep(1)] YP_009168843.1 |  | Hypothetical protein | 4.45 | 17731.69 | AST93237.1 |
| 14 | 8703 | 9317 | + | ATG | TGA | 204 | 9/00E-132 | 93 | Hypothetical protein [Escherichia phage ST2] ASH99388.1 |  | Hypothetical protein | 8.06 | 20902.93 | AST93238.1 |
| 15 | 9317 | 9676 | + | ATG | TGA | 119 | 1/00E-75 | 93 | Hypothetical protein G_16 [Escherichia phage G AB-2017] AQN31773.1 |  | Hypothetical protein | 7.94 | 12968.06 | AST93239.1 |
| 16 | 9673 | 10068 | + | ATG | TGA | 131 | 3/00E-89 | 96 | Putative tail protein [Escherichia phage L AB-2017] AQN31838.1 | Pfam04883 | Putative tail protein | 9.85 | 14550.49 | AST93240.1 |
| 17 | 10065 | 10481 | + | ATG | TGA | 138 | 2/00E-94 | 97 | Hypothetical protein [Escherichia phage K1-dep(4)] YP_009168798.1 | Pfm13554 | Hypothetical protein | 4.85 | 15158.32 | AST93241.1 |
| 18 | 10484 | 11650 | + | ATG | TAA | 388 | 0.0 | 99 | Putative tail protein [Escherichia phage K1-dep(1)] YP_009168848.1 |  | Putative tail protein | 4.82 | 40792.43 | AST93242.1 |
| 19 | 11679 | 12233 | _ | ATG | TAA | 184 | 2/00E-115 | 90 | Hypothetical protein [Escherichia phage K1-ind(3)] ADA82463.1 |  | Hypothetical protein | 5.15 | 20112.87 | AST93243.1 |
| 20 | 12233 | 12721 | _ | ATG | TAA | 162 | 2/00E-108 | 93 | Acid phosphatase [Escherichia phage ST2] ASH99382.1 | Cd07502,PHA02530, pfam03767, COG1778, smart00775, TIGR01675 | Acid phosphatase | 5.52 | 18563.98 | AST93244.1 |
| 21 | 12718 | 13848 | _ | ATG | TGA | 376 | 0.0 | 98 | Calcineurin-like phosphoesterase superfamily domain protein [Escherichia phage L AB-2017] AQN31833.1 |  | Calcineurin-like phosphoesterase superfamily domain protein | 8.44 | 42374.58 | AST93245.1 |
| 22 | 13887 | 14078 | _ | ATG | TAG | 63 | 1/00E-24 | 71 | Hypothetical protein [Escherichia phage K1-dep(4)] YP_009168803.1 |  | Hypothetical protein | 9.86 | 7404.16 | AST93246.1 |
| 23 | 14257 | 14673 | + | ATG | TAG | 138 | 8/00E-89 | 92 | Hypothetical protein [Salmonella phage Ent1] YP_007010478.1 |  | Hypothetical protein | 4.90 | 15899.11 | AST93247.1 |
| 24 | 14679 | 15038 | + | TTG | TGA | 119 | 3/00E-73 | 85 | Hypothetical protein LPSE_00065 [Salmonella phage LPSE1] APU03017.1 |  | Hypothetical protein | 5.13 | 13601.40 | AST93248.1 |
| 25 | 15031 | 17370 | + | ATG | TAA | 779 | 0.0 | 80 | Putative tail tape measure protein [Salmonella phage SETP7] YP_008767197.1 | COG3941 | Putative tape measure protein | 4.82 | 83169.89 | AST93249.1 |
| 26 | 17370 | 18788 | + | ATG | TAA | 472 | 0.0 | 66 | Hypothetical protein [Escherichia phage K1-ind(2)] ADA82421.1 |  | Hypothetical protein | 4.58 | 50238.96 | AST93250.1 |
| 27 | 18792 | 19307 | + | ATG | TGA | 171 | 2/00E-116 | 96 | Hypothetical protein G_4 [Escherichia phage G AB-2017] AQN31761.1 | Pfam08875 | Hypothetical protein | 4.43 | 19062.56 | AST93251.1 |
| 28 | 19304 | 19669 | + | ATG | TAA | 121 | 4/00E-86 | 100 | Hypothetical protein [Escherichia phage K1-dep(1)] YP_009168858.1 |  | Hypothetical protein | 6.50 | 14064.87 | AST93252.1 |
| 29 | 19660 | 22227 | + | TTG | TAA | 855 | 0.0 | 82 | Putative tail protein [Escherichia phage ST2] ASH99373.1 |  | Putative tail protein | 4.61 | 81096.76 | AST93253.1 |
| 30 | 22240 | 24225 | + | ATG | TAA | 661 | 0.0 | 85 | Phage tail fibers [Escherichia phage LM33_P1] YP_009324518.1 |  | Phage tail fibers | 5.37 | 56650.55 | AST93254.1 |
| 31 | 24254 | 24391 | _ | ATG | TAG | 45 | 1/00E-21 | 100 | Exonuclease subunit SbcD [Escherichia phage L AB-2017] AQN31941.1 | PRK10966, pfam13600 | Exonuclease subunit SbcD | 5.26 | 5216.09 | AST93255.1 |
| 32 | 24388 | 24903 | _ | ATG | TGA | 171 | 2/00E-31 | 49 | Hypothetical protein APCEc03_001 [Escherichia phage APCEc03] AKO61402.1 |  | Hypothetical protein | 7.62 | 19462.26 | AST93256.1 |
| 33 | 24900 | 26324 | _ | ATG | TGA | 474 | 0.0 | 99 | Helicase [Escherichia phage G AB-2017] AQN31823.1 | COG0553, pfam00176, smart00487, cd00046 | Helicase | 8.66 | 53506.95 | AST93257.1 |
| 34 | 26317 | 27246 | _ | ATG | TAA | 309 | 0.0 | 93 | Putative C-specific methylase [Escherichia phage K1-ind(3)] ADA82477.1 | COG0270, cd00315, pfam00145, TIGR00675 | Putative C-specific methylase | 6.13 | 34115.30 | AST93258.1 |
| 35 | 27243 | 27434 | _ | ATG | TGA | 63 | 4/00E-37 | 98 | Hypothetical protein PVPSE2_24 [Salmonella phage vB_SenS_PVP-SE2] AST15505.1 |  | Hypothetical protein | 9.16 | 7087.23 | AST93259.1 |
| 36 | 27465 | 27752 | _ | ATG | TAG | 95 | 2/00E-60 | 93 | VRR-NUC domain protein [Escherichia phage ST2] ASH99367.1 | Smart00990 | VRR-NUC domain protein | 9.47 | 10735.37 | AST93260.1 |
| 37 | 27739 | 27837 | - | ATG | TGA | 32 | 8/00E-10 | 78 | Hypothetical protein (salmonella phage vB_SenS_AG11) AFO12436.1 |  | Hypothetical protein | 6.53 | 3939.51 | AST93261.1 |
| 38 | 27834 | 27965 | _ | ATG | TGA | 43 | 7/00E-20 | 91 | Hypothetical protein G_62 [Escherichia phage G AB-2017] AQN31819.1 |  | Hypothetical protein | 5.38 | 4659.70 | AST93262.1 |
| 39 | 27955 | 30207 | _ | ATG | TAA | 750 | 0.0 | 94 | Putative DNA polymerase [Escherichia phage K1-ind(3)] ADA82478.1 | Cd08642, smart00482, PRK14975, pfam00476, TIGR00593, COG0749, pfam01612 | Putative DNA polymerase | 7.84 | 84952.75 | AST93263.1 |
| 40 | 30204 | 30482 | _ | ATG | TGA | 92 | 2/00E-27 | 58 | Hypothetical protein SU10_055 [Escherichia phage vB_EcoP_SU10] YP_009152906.1 |  | Hypothetical protein | 8.96 | 11329.89 | AST93264.1 |
| 41 | 30542 | 31171 | _ | ATG | TAA | 209 | 7/00E-150 | 99 | Hypothetical protein [Escherichia phage ST2] ASH99364.1 | Pfam10991 | Hypothetical protein | 4.89 | 23494.22 | AST93265.1 |
| 42 | 31250 | 31453 | - | ATG | TAG | 67 | - | - | - |  | Hypothetical protein | 4.40 | 8158.32 | AST93266.1 |
| 43 | 31450 | 32691 | _ | ATG | TGA | 413 | 0.0 | 94 | PD-(D/E)XK nuclease superfamily protein [Escherichia phage G AB-2017] AQN31815.1 | Pfam10926 | PD-(D/E)XK nuclease superfamily protein | 6.31 | 45995.11 | AST93267.1 |
| 44 | 32688 | 33035 | _ | ATG | TGA | 115 | 7/00E-77 | 97 | Hypothetical protein STP03_062 [Salmonella phage STP03] APM00316.1 |  | Hypothetical protein | 9.43 | 13385.21 | AST93268.1 |
| 45 | 33032 | 33304 | _ | ATG | TGA | 90 | 2/00E-60 | 96 | Hypothetical protein Jersey_47 [Salmonella phage Jersey] YP_008239756.1 |  | Hypothetical protein | 9.25 | 10852.43 | AST93269.1 |
| 46 | 33347 | 33850 | _ | ATG | TAA | 167 | 4/00E-69 | 72 | Hypothetical protein PVPSE2_18 [Salmonella phage vB_SenS_PVP-SE2] AST15477.1 |  | Hypothetical protein | 4.39 | 18908.11 | AST93270.1 |
| 47 | 33873 | 34100 | _ | ATG | TAA | 75 | 5/00E-41 | 91 | Hypothetical protein O25BPHAGE1_P1_GP4 [Escherichia phage LM33_P1] YP_009324478.1 |  | Hypothetical protein | 4.59 | 8160.20 | AST93271.1 |
| 48 | 34222 | 34437 | + | ATG | TGA | 71 | 9/00E-41 | 99 | Transcriptional repressor DicA [Escherichia phage L AB-2017] AQN31878.1 | Cd00093, PRK09706, smart00530, pfam01381 | Transcriptional repressor DicA | 8.25 | 7730.96 | AST93272.1 |
| 49 | 34451 | 34648 | _ | ATG | TAA | 65 | 6/00E-39 | 98 | Hypothetical protein [Escherichia phage K1-dep(4)] YP_009168823.1 |  | Hypothetical protein | 9.48 | 7522.63 | AST93273.1 |
| 50 | 34645 | 36906 | _ | ATG | TGA | 753 | 0.0 | 98 | Putative replicative helicase/primease [Escherichia phage G AB-2017] AQN31809.1 | Pfam13481, cd01125 | Putative replicative helicase/primease | 5.39 | 84768.48 | AST93274.1 |
| 51 | 36903 | 37031 | _ | ATG | TGA | 42 | 7/00E-21 | 98 | Hypothetical protein G_51 [Escherichia phage G AB-2017] AQN31808.1 |  | Hypothetical protein | 11.41 | 5026.04 | AST93275.1 |
| 52 | 37082 | 37315 | _ | ATG | TAG | 77 | 1/00E-11 | 45 | Hypothetical protein G_50 [Escherichia phage G AB-2017] AQN31807.1 |  | Hypothetical protein | 6.18 | 9035.29 | AST93276.1 |
| 53 | 37312 | 37500 | _ | ATG | TGA | 62 | 5/00E-37 | 98 | Helix-turn-helix domain protein [Escherichia phage G AB-2017] AQN31806.1 | Pfam12728, cd00093, COG1476, TIGR01764, smart00530 | Helix-turn-helix protein | 9.92 | 7354.60 | AST93277.1 |
| 54 | 37974 | 38165 | + | ATG | TGA | 63 | 2/00E-18 | 63 | Hypothetical protein G_48 [Escherichia phage G AB-2017] AQN31805.1 |  | Hypothetical protein | 9.35 | 7251.33 | AST93278.1 |
| 55 | 38165 | 38383 | + | ATG | TAA | 72 | - | - | - |  | Hypothetical protein | 5.24 | 7857.90 | AST93279.1 |
| 56 | 38426 | 38620 | + | ATG | TGA | 64 | 1/00E-35 | 92 | Hypothetical protein SP101_00225 [Salmonella phage FSL SP-101] AGF87748.1 |  | Hypothetical protein | 9.84 | 7552.71 | AST93280.1 |
| 57 | 38617 | 38760 | + | ATG | TGA | 47 | 2/00E-23 | 96 | Hypothetical protein P_47 [Escherichia phage P AB-2017] AQN31985.1 |  | Hypothetical protein | 10.61 | 5520.47 | AST93281.1 |
| 58 | 38764 | 38952 | + | ATG | TAA | 62 | 5/00E-10 | 50 | [Hypothetical protein LPST10_00021 [Salmonella phage LPST10] ARK07753.1](https://www.ncbi.nlm.nih.gov/protein/1184850218?report=genbank&log$=prottop&blast_rank=14&RID=VYXC5WYM016) | Pfam04448 | Hypothetical protein | 4.63 | 7086.09 | AST93282.1 |
| 59 | 39173 | 39340 | + | ATG | TAA | 55 | - | - | - |  | Hypothetical protein | 9.30 | 6557.36 | AST93283.1 |
| 60 | 39330 | 39782 | + | ATG | TGA | 150 | 4/00E-21 | 57 | Hypothetical protein [Salmonella phage BPS11Q3] YP_009322833.1 |  | Hypothetical protein | 5.27 | 17293.29 | AST93284.1 |
| 61 | 39779 | 39982 | + | ATG | TAA | 67 | 1/00E-41 | 99 | Hypothetical protein BPS11Q3_25 [Salmonella phage BPS11Q3] YP_009322832.1 |  | Hypothetical protein | 7.81 | 7338.39 | AST93285.1 |
| 62 | 39984 | 40166 | + | ATG | TGA | 60 | 7/00E-31 | 88 | Hypothetical protein G_39 [Escherichia phage G AB-2017] AQN31796.1 |  | Hypothetical protein | 4.25 | 6874.70 | AST93286.1 |
| 63 | 40169 | 40414 | + | ATG | TGA | 81 | 3/00E-15 | 42 | Hypothetical protein [Salmonella phage IME207] YP_009322750.1 |  | Hypothetical protein | 9.43 | 9682.92 | AST93287.1 |
| 64 | 40411 | 40500 | + | ATG | TGA | 29 | 1/00E-15 | 89 | Hypothetical protein [Salmonella phage IME207] YP_009322751.1 |  | Hypothetical protein | 3.77 | 3287.92 | AST93288.1 |
| 65 | 40497 | 40790 | + | ATG | TAA | 97 | 5/00E-62 | 95 | Hypothetical protein [Escherichia phage K1ind1] ADA82387.1 |  | Hypothetical protein | 10.84 | 11237.02 | AST93289.1 |
| 66 | 40866 | 41174 | + | TTG | TGA | 102 | 7/00E-65 | 99 | Putative holin-like, class II [Escherichia phage K1-dep(4)] YP_009168828.1 |  | Putative holin-like, class II | 6.50 | 2452.77 | AST93290.1 |
| 67 | 41167 | 41439 | + | ATG | TAA | 90 | 1/00E-57 | 96 | Putative holin-like, class I [Escherichia phage K1-ind(3)] ADA82487.1 |  | Putative holin-like, class I | 8.77 | 10109.91 | AST93291.1 |
| 68 | 41444 | 41902 | + | TTG | TGA | 152 | 9/00E-93 | 93 | Lysozyme [Escherichia phage G AB-2017] AQN31792.1 | Cd00737, COG3772, pfam00959 | Lysozyme/  endolysin | 10.27 | 10313.89 | AST93292.1 |
| 69 | 42184 | 42432 | + | ATG | TGA | 82 | 4/00E-26 | 100 | Hypothetical protein G_34 [Escherichia phage G AB-2017] AQN31791.1 |  | Hypothetical protein | 6.16 | 9041.61 | AST93293.1 |
| 70 | 42429 | 42626 | + | ATG | TGA | 65 | - | - | - |  | Hypothetical protein | 4.35 | 7264.20 | AST93294.1 |
| 71 | 42623 | 42784 | + | ATG | TGA | 53 | 6/00E-29 | 92 | [Hypothetical protein P_37 [Escherichia phage P AB-2017] AQN31975.1](https://blast.ncbi.nlm.nih.gov/Blast.cgi#alnHdr_1145307300) | Pfam10930 | Hypothetical protein | 10.27 | 6298.23 | AST93295.1 |
| 72 | 42777 | 42962 | + | ATG | TAA | 61 | 1/00E-33 | 95 | Hypothetical protein [Escherichia phage ST2] ASH99336.1 |  | Hypothetical protein | 4.94 | 6991.98 | AST93296.1 |
| 73 | 42955 | 43242 | + | ATG | TAA | 95 | 1/00E-19 | 91 | Hypothetical protein [Escherichia phage ST2] ASH99335.1 |  | Hypothetical protein | 4.12 | 10699.11 | AST93297.1 |
| 74 | 43235 | 43420 | + | ATG | TGA | 61 | 7/00E-34 | 97 | Hypothetical protein L_30 [Escherichia phage L AB-2017] AQN31855.1 |  | Hypothetical protein | 4.82 | 6842.80 | AST93298.1 |
| 75 | 43508 | 43678 | + | ATG | TGA | 56 | 6/00E-35 | 100 | Hypothetical protein P_34 [Escherichia phage P AB-2017] AQN31972.1 |  | Hypothetical protein | 7.96 | 6676.55 | AST93299.1 |
| 76 | 43675 | 43911 | + | ATG | TGA | 78 | 3/00E-50 | 99 | Hypothetical protein [Escherichia phage ST2] ASH99331.1 | Pfam06322 | Hypothetical protein | 9.10 | 8890.26 | AST93300.1 |
| 77 | 43908 | 44090 | + | ATG | TGA | 60 | 5/00E-31 | 90 | Hypothetical protein [Salmonella phage vB_SenS-Ent3] YP_009035197.1 |  | Hypothetical protein | 9.69 | 6859.34 | AST93301.1 |
| 78 | 44295 | 44819 | + | ATG | TAA | 174 | 2/00E-115 | 96 | Putative terminase small subunit [Escherichia phage ST2] ASH99402.1 |  | Putative terminase small subunit | 6.76 | 19407.12 | AST93302.1 |

^1^amino acids, ^2^ Molecular weight, ^3^ pH isoelectric.

**Supplementary Table S4. Predicted conserved promoter sequences of the VB_EcoS-Golestan genome using PHIRE.**

| **Number** | **Name** | **Promoter sequence** | **Beginning** | **End** | **Orientation** |
| --- | --- | --- | --- | --- | --- |
| 1 | Before ORF3 | TTATAGTATGCTATTAACTT | 2775 | 2794 | Forward |
| 2 | Before ORF7 | TAATAGGGTACTATTCATTA | 4898 | 4879 | Backward |
| 3 | Before ORF23 | TAATAGCGTACTATTCACTT | 14134 | 14115 | Backward |
| 4 | Before ORF48 | TAATAGCGAACTATTCACAT | 34133 | 34152 | Forward |
| 5 | Before ORF48 | TAATATGGTACTATTCACAT | 34177 | 34196 | Forward |
| 6 | Before ORF54 | TAATAGGATACTATTCACTT | 37926 | 37945 | Forward |
| 7 | Before ORF59 | GAATAGGCTACTATTCATTT | 39128 | 39147 | Forward |

**Supplementary Table S5.** Rho-factor-independent terminators of VB_EcoS-Golestan

| **Number** | **Position** | **Strand** | **Sequence** | **ΔG (kcal/mol)** |
| --- | --- | --- | --- | --- |
| T01 | 2746 | + | GCCCCGAAAGGGGCTTTCTTATAGTA | -14.30 |
| T02 | 4312 | + | GAGGCCCCGAAAGGGGCTTTaTTTTTATATGAAC | -17.40 |
| T03 | 5179 | + | GCCCCGCTTTAAGGCGGGGCTTTACTTATCGA | -17.70 |
| T04 | 7572 | + | GAGGGACTTCGGTCCCTCTTTTCATTTACT | -16.30 |
| T05 | 7962 | - | CGGCCCCGAAGGGCCGTTGTATTAAGAC | -12.20 |
| T06 | 11645 | - | GGCCCCGAAAGGGGCCTTAGTTTTATGC | -17.60 |
| T07 | 11646 | + | GGCCCCTTTCGGGGCCTTTTTCTTACAA | -13.80 |
| T08 | 22401 | + | CCGTGTTGCGCGACGCGGgTTTTTCTCCGGT | -10.20 |
| T09 | 24220 | - | GGCCCCGTTTGGGGCCTTAGTTTTAAGC | -13.50 |
| T10 | 30493 | - | GGCCCTTCATTGGGCCTTTTTACTAAGG | -11.30 |
| T11 | 34418 | + | GGGGCTTGACGCCCCTTTCTTTTTATT | -10.50 |
| T12 | 37061 | + | CCCCGACGTTCTAGCGTTGGGGTTTCTTTTGCCT | -14.80 |
| T13 | 44183 | + | GCCCTCTACGGAGGGCTTTTCTGTACAT | -14.60 |


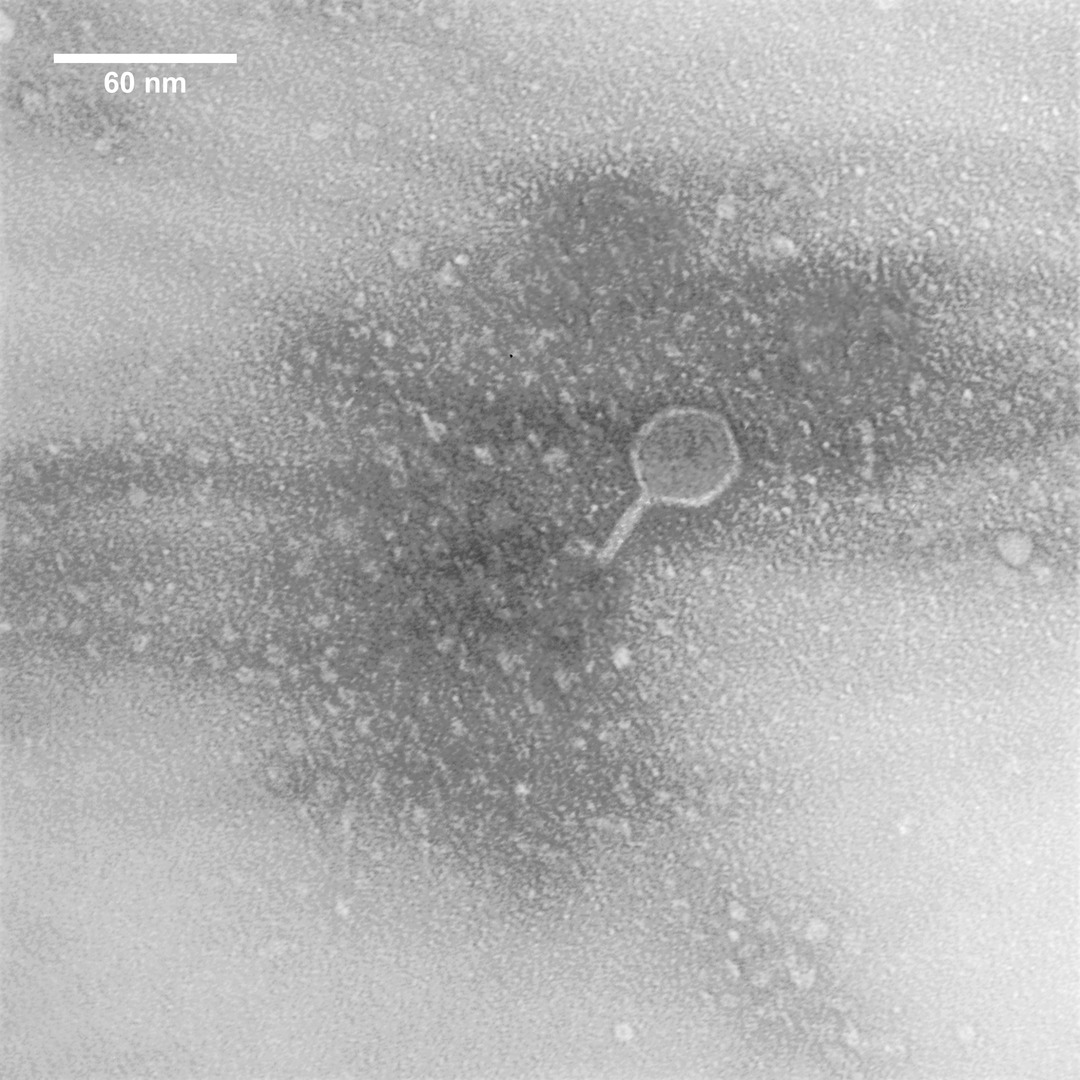

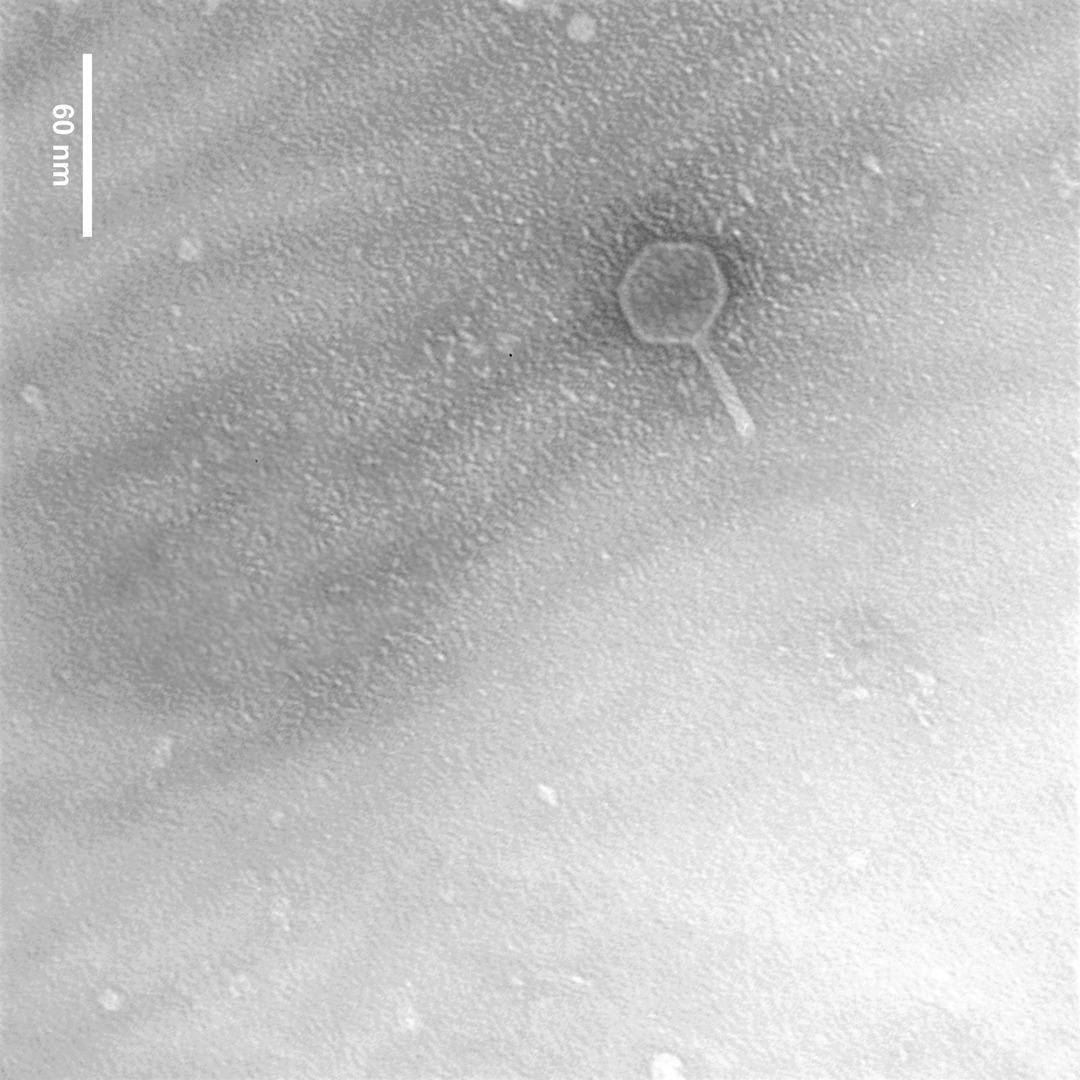


**Supplementary** **Figure S1.** Additional images of transmission electron micrograph of the phage VB_EcoS-Golestan.


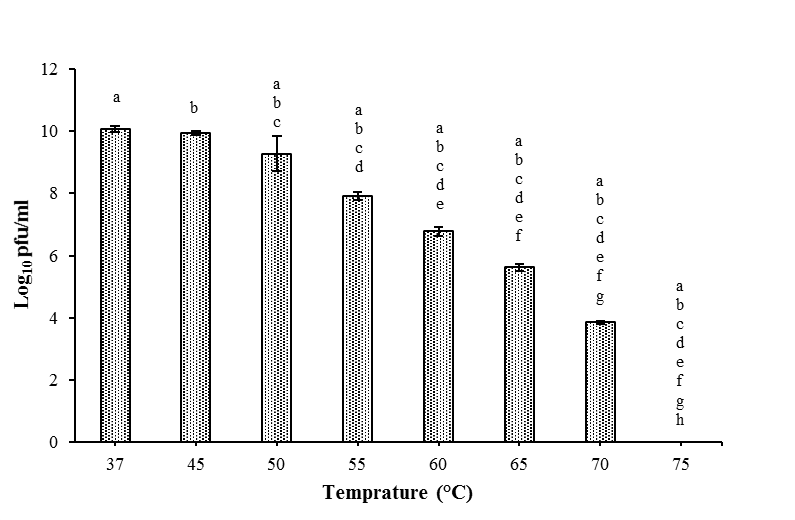


**a**

**b**


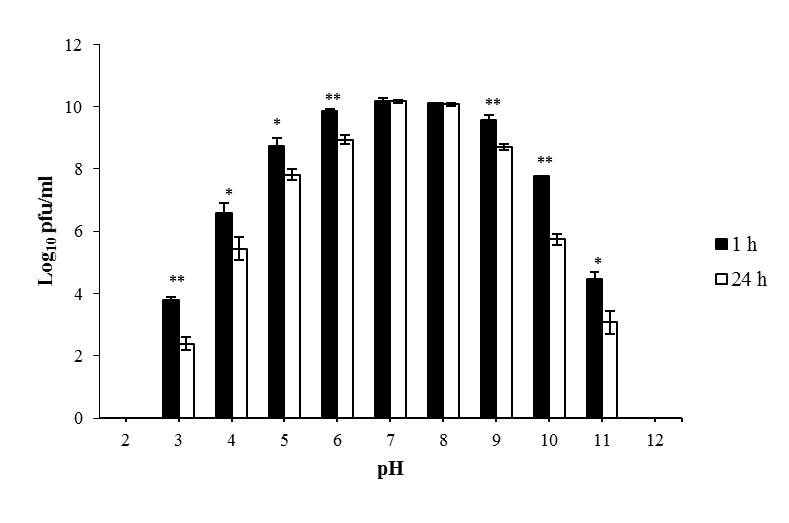


**Supplementary** **Figure S2**. Temperature stability of the phage VB_EcoS-Golestan at different temperatures for 1h. The difference in the titer of the virus in columns with the same letters were significant (One-way ANOVA Repeated measures, P ≤ 0.05) (a). Stability of the phage treated with different pH values for 1h and 24h at 37°C (b). Significant differences in phage titers at 1h compare to 24h in different pH values. (T-test,* = *P* ≤ 0.05, ** = *P* ≤ 0.01)


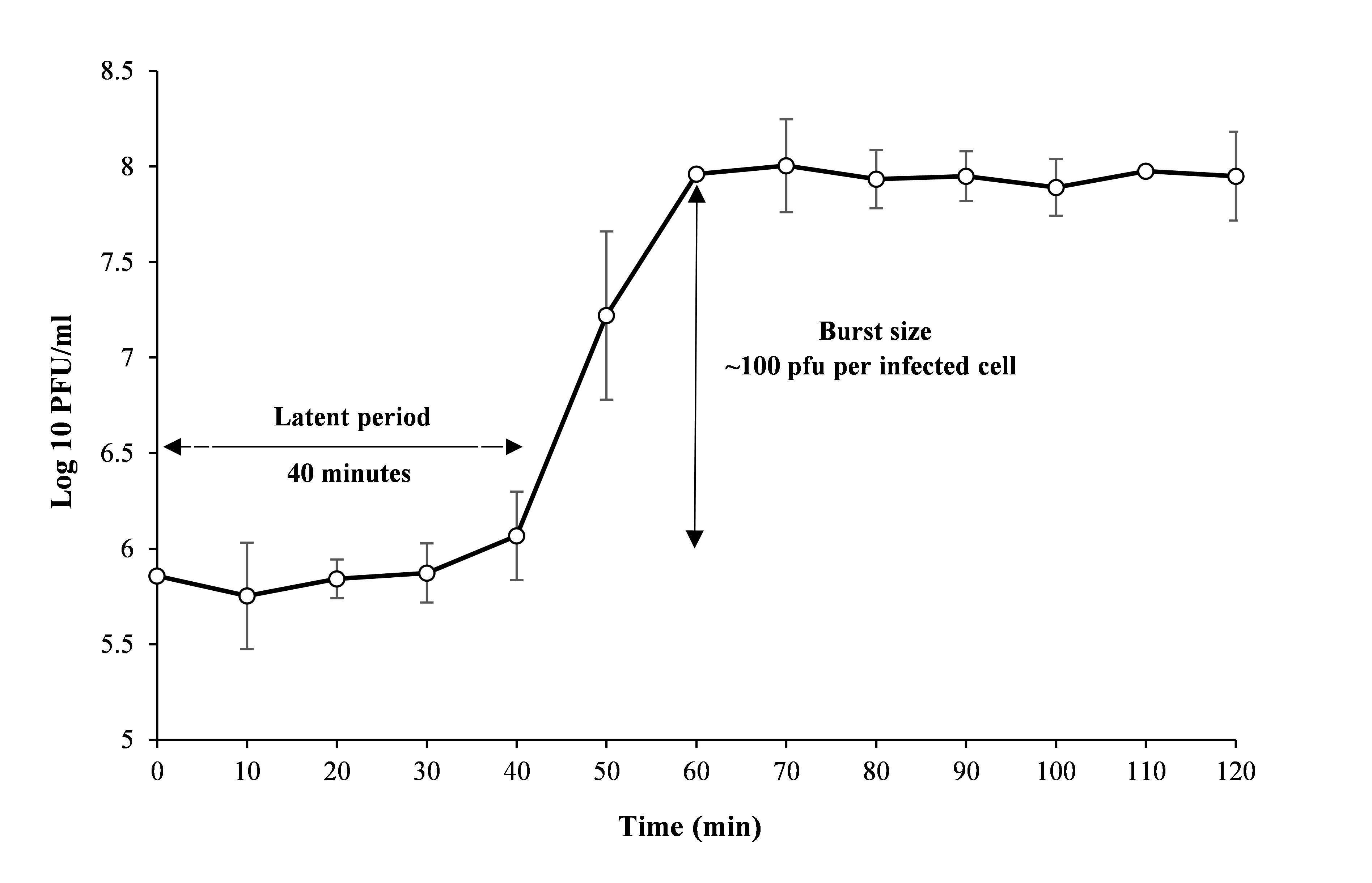


**Supplementary Figure S3**. One step growth curve of phage VB_EcoS-Golestan.


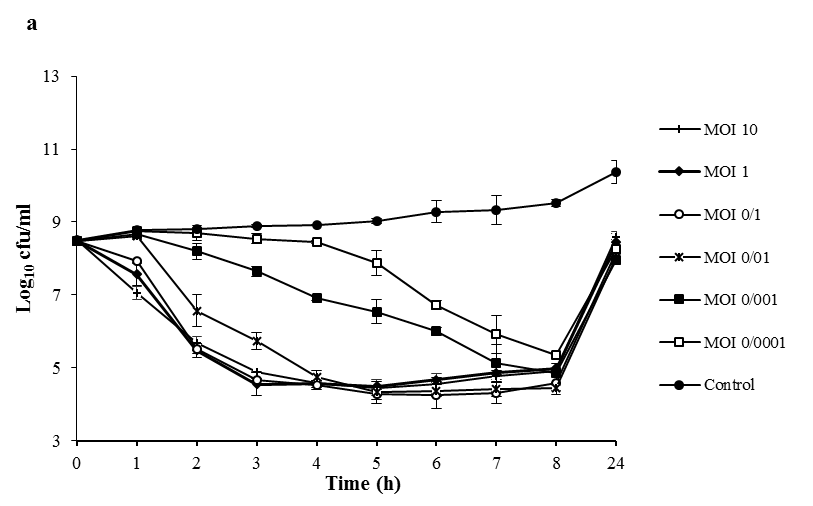


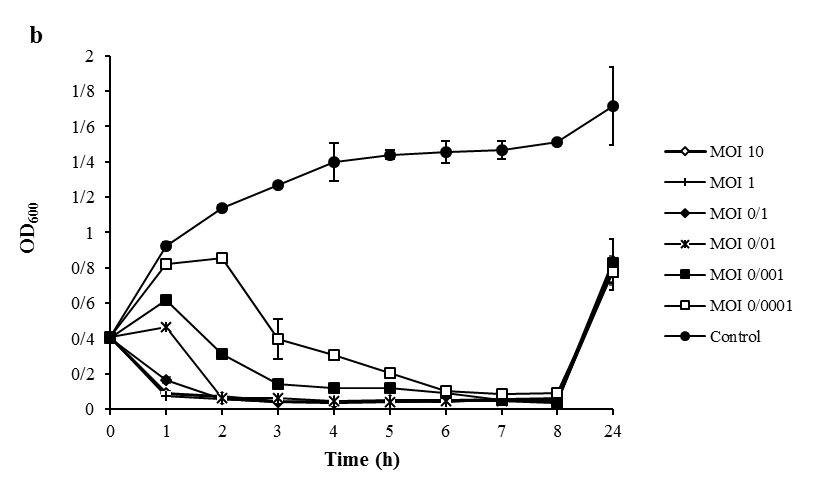


**Supplementary Figure S4**. The bacteriolytic activity of the VB_EcoS-Golestan in different MOIs against *E. coli* according to cell count (a) and optical density (OD_600_) (b). All values represent the means of three determinations ±SD.


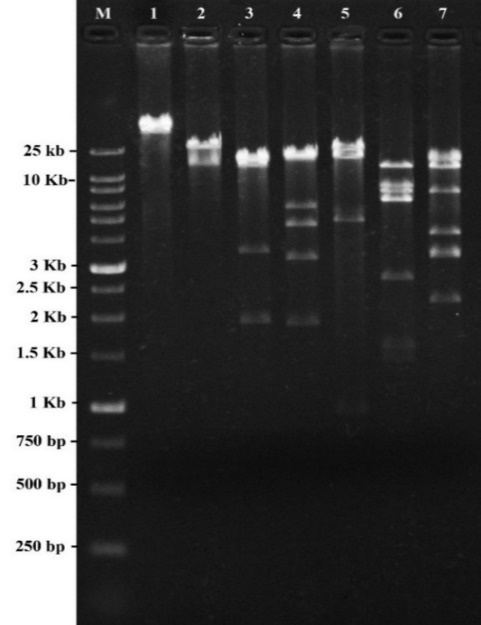


**Supplementary Figure S5**. Restriction fragments analysis of the phage vB_EcoS-Golestan. Untreated phage genomic DNA (lane 1) and digested with restriction enzymes BamHI (lane 2), NdeI (lane 3), HindIII (lane 4), EcoRI (lane 5), EcoRV (lane 6), and PstI (lane 7). Lane M, 25 Kb DNA marker (Cinacolon, Iran).
